# Prolonged indoleamine 2,3-dioxygenase-2 activity and associated cellular stress in post-acute sequelae of SARS-CoV-2 infection

**DOI:** 10.1016/j.ebiom.2023.104729

**Published:** 2023-07-26

**Authors:** Lihui Guo, Brent Appelman, Kirsten Mooij-Kalverda, Riekelt H. Houtkooper, Michel van Weeghel, Frédéric M. Vaz, Annemiek Dijkhuis, Tamara Dekker, Barbara S. Smids, Jan Willem Duitman, Marianna Bugiani, Paul Brinkman, Jonne J. Sikkens, H.A. Ayesha Lavell, Rob C.I. Wüst, Michèle van Vugt, René Lutter, Amsterdam UMC COVID-19 Biobank study Group, M.A. van Agtmael, A.G. Algera, B. Appelman, F.E.H.P. van Baarle, M. Beudel, H.J. Bogaard, M. Bomers, P.I. Bonta, L.D.J. Bos, M. Botta, J. de Brabander, G.J. de Bree, S. de Bruin, M. Bugiani, E.B. Bulle, O. Chouchane, A.P.M. Cloherty, D. Buis, M.C.F.J. de Rotte, M. Dijkstra, D.A. Dongelmans, R.W.G. Dujardin, P.E. Elbers, L.M. Fleuren, S.E. Geerlings, T.B.H. Geijtenbeek, A.R.J. Girbes, A. Goorhuis, M.P. Grobusch, L.A. Hagens, J. Hamann, V.C. Harris, R. Hemke, S.M. Hermans, L.M.A. Heunks, M.W. Hollmann, J. Horn, J.W. Hovius, M.D. de Jong, R. Koing, E.H.T. Lim, N. van Mourik, J.F. Nellen, E.J. Nossent, F. Paulus, E. Peters, D. Piña-Fuentes, T. van der Poll, B. Preckel, J.M. Prins, S.J. Raasveld, T.D.Y. Reijnders, M. Schinkel, F.A.P. Schrauwen, M.J. Schultz, A.R. Schuurman, J. Schuurmans, K. Sigaloff, M.A. Slim, P. Smeele, M.R. Smit, C. Stijnis, W. Stilma, C.E. Teunissen, P. Thoral, A.M. Tsonas, P.R. Tuinman, M. van der Valk, D.P. Veelo, C. Volleman, H. de Vries, L.A. van Vught, M. van Vugt, D. Wouters, A.H. Zwinderman, M.C. Brouwer, W.J. Wiersinga, A.P.J. Vlaar, D. van de Beek

**Affiliations:** a Department Experimental Immunology, Amsterdam Infection and Immunity Center, Amsterdam University Medical Centers (UMC), location Academic Medical Center (AMC), University of Amsterdam, Meibergdreef 9, 1105 AZ Amsterdam, the Netherlands; b Center for Experimental and Molecular Medicine, Amsterdam UMC, Amsterdam Institute for Infection and Immunity, location AMC, University of Amsterdam, Meibergdreef 9, 1105 AZ Amsterdam, the Netherlands; c Department Pulmonary Medicine, Amsterdam UMC, location AMC, University of Amsterdam, Meibergdreef 9, 1105 AZ Amsterdam, the Netherlands; d Laboratory Genetic Metabolic Diseases, Amsterdam UMC, location AMC, University of Amsterdam, Meibergdreef 9, 1105 AZ Amsterdam, the Netherlands; e Amsterdam Gastroenterology Endocrinology and Metabolism Institute, Amsterdam, the Netherlands; f Amsterdam Cardiovascular Sciences Institute, Amsterdam, the Netherlands; g Core Facility Metabolomics, Amsterdam UMC, location AMC, University of Amsterdam, Meibergdreef 9, 1105 AZ Amsterdam, the Netherlands; h Department of Clinical Chemistry and Pediatrics, Laboratory Genetic Metabolic Diseases, Emma Children's Hospital, Amsterdam UMC, location AMC, University of Amsterdam, Meibergdreef 9, 1105 AZ Amsterdam, the Netherlands; i Amsterdam Gastroenterology Endocrinology Metabolism, Inborn Errors of Metabolism, Amsterdam, the Netherlands; j Department of Pathology, Amsterdam UMC, Vrije Universiteit Amsterdam, Amsterdam, the Netherlands; k Department of Internal Medicine, Amsterdam UMC, Vrije Universiteit Amsterdam, Amsterdam Institute for Infection and Immunity, Amsterdam, the Netherlands; l Laboratory for Myology, Department of Human Movement Sciences, Faculty of Behavioural and Movement Sciences, Vrije Universiteit Amsterdam, Amsterdam Movement Sciences, Amsterdam, the Netherlands; m Division of Infectious Diseases, Department of Internal Medicine, Amsterdam UMC, location AMC, University of Amsterdam, Meibergdreef 9, 1105 AZ Amsterdam, the Netherlands

**Keywords:** IDO2, PASC, Autophagy, Apoptosis, Kynurenine, Mitochondrial_activity

## Abstract

**Background:**

Post-acute sequela of SARS-CoV-2 infection (PASC) encompass fatigue, post-exertional malaise and cognitive problems. The abundant expression of the tryptophan-catabolizing enzyme indoleamine 2,3-dioxygenase-2 (IDO2) in fatal/severe COVID-19, led us to determine, in an exploratory observational study, whether IDO2 is expressed and active in PASC, and may correlate with pathophysiology.

**Methods:**

Plasma or serum, and peripheral blood mononuclear cells (PBMC) were obtained from well-characterized PASC patients and SARS-CoV-2-infected individuals without PASC. We assessed tryptophan and its degradation products by UPLC-MS/MS. IDO2 activity, its potential consequences, and the involvement of the aryl hydrocarbon receptor (AHR) in IDO2 expression were determined in PBMC from another PASC cohort by immunohistochemistry (IHC) for IDO2, IDO1, AHR, kynurenine metabolites, autophagy, and apoptosis. These PBMC were also analyzed by metabolomics and for mitochondrial functioning by respirometry. IHC was also performed on autopsy brain material from two PASC patients.

**Findings:**

IDO2 is expressed and active in PBMC from PASC patients, as well as in brain tissue, long after SARS-CoV-2 infection. This is paralleled by autophagy, and in blood cells by reduced mitochondrial functioning, reduced intracellular levels of amino acids and Krebs cycle-related compounds. IDO2 expression and activity is triggered by SARS-CoV-2-infection, but the severity of SARS-CoV-2-induced pathology appears related to the generated specific kynurenine metabolites. *Ex vivo*, IDO2 expression and autophagy can be halted by an AHR antagonist.

**Interpretation:**

SARS-CoV-2 infection triggers long-lasting IDO2 expression, which can be halted by an AHR antagonist. The specific kynurenine catabolites may relate to SARS-CoV-2-induced symptoms and pathology.

**Funding:**

None.

**Research in context**

**Evidence before this study**

Earlier studies indicated that the degradation of tryptophan to kynurenine and further breakdown products was enhanced in SARS-CoV-2 infected individuals, likely caused by the interferon-induced tryptophan-catabolizing enzyme indoleamine 2,3-dioxygenase (IDO)1, which typically is induced by viral infections. Rather than IDO1, our immunohistochemical analyses of lung, heart, brain and blood cells from patients with fatal/severe COVID-19 showed extensive expression and activity of the otherwise rarely expressed IDO2. This was associated with reduced systemic tryptophan and enhanced breakdown products (among others, kynurenine and the neuro-/cytotoxic quinolinic acid and 3OH-kynurenine) and, spatially, with autophagy and apoptosis. This led us to study IDO2 expression in patients with post-acute sequelae of SARS-CoV-2 infection (PASC).

**Added value of this study**

IDO2 is expressed in blood cells from PASC patients and manifests itself long after the initial infection by SARS-CoV-2. IDO2 expression is driven by the aryl hydrocarbon receptor (AHR) and coincides with an altered cellular metabolism, reflected by attenuated mitochondrial functioning and autophagy. SARS-CoV-2 infection induces IDO2 expression, but the resulting pathology appears related to the specific kynurenine breakdown products that are generated. In contrast to fatal/severe COVID-19 we found marked autophagy and limited apoptosis in PASC. This was confirmed in autopsy brain tissue from PASC patients. All PASC patients experienced an increase in plasma xanthurenic acid levels, while only some patients exhibited significantly elevated levels of 3-hydroxy anthranilic acid.

**Implications of all the available evidence**

Together these findings imply the AHR-IDO2-kynurenine pathway in SARS-CoV-2-induced pathology and that of PASC in particular. Further studies into IDO2 activity and the down-stream kynurenine products at the cellular level in a larger PASC cohort, and its association with quantitative parameters of symptoms would substantiate these finding. An explorative trial in PASC to interfere with the AHR-IDO2 pathway is warranted. As there are no potent inhibitors of IDO2 available for use in humans, an AHR antagonist is a likely therapeutic option.

## Introduction

Coronavirus disease 2019 (COVID-19), which is triggered by infection with the severe acute respiratory syndrome coronavirus 2 (SARS-CoV-2), manifests itself in various ways, ranging from a mild to fatal course of disease, and from full recovery to marked and various long-lasting symptoms.^1^ When such long-lasting symptoms persist for at least three months after the initial symptoms, this was initially referred to as long-COVID, later renamed as Post-Acute Sequelae of SARS-CoV-2 infection (PASC).^2^ The symptoms of PASC vary in intensity, are heterogeneous, and significantly attenuate quality of life. The most disabling symptoms are severe fatigue, cognitive dysfunction and post-exertional malaise.^3^^,^^4^ As hospitalization in itself can induce various forms of fatigue, such as cachexia and sarcopenia, it is important to distinguish these from PASC symptoms.^5^ The nature of the pathological process (es) leading to PASC is (are) still unknown.

Recently, we showed that the otherwise rarely expressed tryptophan-catabolizing enzyme indoleamine 2,3-dioxygenase-2 (IDO2) was abundantly expressed and enzymatically active in lung, heart and brain, and also monocytes and lymphocytes of patients with fatal COVID-19.^6^ IDO2, like its isotype IDO1, degrades the essential amino acid tryptophan, which can affect protein synthesis and lead to autophagy.^7^^,^^8^ IDO2 generates formylkynurenine, which can be metabolized further to kynurenine and downstream cyto- and neurotoxic metabolites, such as 3OH-kynurenine, 3OH-anthranilic acid and quinolinic acid, potentially leading to apoptosis.^9^, ^10^, ^11^ In tissue from fatal COVID-19 patients, IDO2 expression, kynurenine metabolites and markers of autophagy and apoptosis closely co-localized, suggestive of IDO2 contributing to the pathophysiology in fatal COVID-19.

IDO2 expression can be induced by a ligand-activated transcription factor, the aryl hydrocarbon receptor (AHR).^12^^,^^13^ Intriguingly, infection with coronavirus species has been shown to activate AHR in mice^14^ and recently also for SARS-CoV-2 in humans.^15^ Indeed, in fatal COVID-19 AHR was localized in the nucleus of IDO2-expressing cells,^6^ indicative of its transcriptional activity, and therefore likely driving IDO2 expression. Together this led us to assess whether IDO2 was also expressed and active in PASC patients and, if so, whether that could be linked to pathophysiological processes.

## Material and methods

### Study participants

For this exploratory observational study we analysed samples from four different study cohorts. Based on our previous study^6^ we aimed to analyse 12–15 samples of each patient group. EDTA plasma samples from PASC patients were provided through the Amsterdam UMC COVID-19 biobank. PASC patients had a minimum duration of symptoms of three months and reported a wide variety of severe debilitating symptoms (mainly fatigue, post-exertional malaise and cognitive problems; overview provided in Supplemental Table S1) markedly affecting their quality of life (information on their health status before SARS-CoV-2 infection was available). The inclusion and exclusion criteria for the PASC patients are identical to those provided in the registration of the MUSCLE-PASC study (https://clinicaltrials.gov/ct2/show/NCT05225688; criteria provided in Supplemental Table S2). None of the PASC patients were hospitalized during SARS-CoV-2 infection, to preclude a bias of PASC symptoms induced by hospitalization in itself.^5^ All PASC patients were screened by two clinicians specialized in PASC (BA and MvV). SARS-CoV-2 infection was determined by PCR or a rapid antigen test (Wantai). None of these patients was vaccinated before infection. EDTA plasma samples from hospitalized SARS-CoV-2-infected patients with no or only mild remaining symptoms, referred to as ‘Recov’, were provided through the Amsterdam UMC COVID-19 biobank. SARS-CoV-2 infection was determined by PCR. Serum samples (no EDTA plasma available) from non-hospitalized SARS-CoV-2-infected participants, not followed up for symptoms and so referred to as ‘healthy’, were provided through the S3 study and Amsterdam UMC COVID-19 biobank. Infection was assessed by PCR or Wantai. None of these controls were vaccinated before infection. PBMC from non-hospitalized PASC patients and SARS-CoV-2-infected participants without remaining symptoms (referred to as ‘healthy (2)’ in Supplemental Figure S1), both PCR confirmed, were obtained from the MUSCLE-PASC study. PASC patients here met the same inclusion and exclusion criteria and symptoms as the PASC cohort above, and had reduced working hours due to PASC. Both PASC patient groups were screened by the same two post-COVID clinicians (BA and MvV). For the Seahorse experiments we also used PBMC from healthy individuals claiming they have not been infected with SARS-CoV-2 before sampling, and were referred to as non-Covid. PBMC were isolated from heparinized blood and separated over Lymphoprep™. An overview of the various cohorts and the determined parameters is given in Supplemental Figure S1. From two PASC patients we obtained autopsy brain material for immunohistochemical analyses. Both patients were diagnosed with PASC by clinicians outside our hospital, following the WHO guidelines, and verified by our pathologist (MB). The first patient was a 52 year old male with a medical history of a stroke (7 years ago) and symptomatic epilepsy (since 6 years). The patient had a SARS-CoV-2 infection four months prior to his death and since then had symptoms of brainfog, difficulty in concentrating and malaise. These symptoms were not present before SARS-CoV-2 infection. The patient died of a status epilepticus. The second patient was a 80 year old male of whom the medical history was not yet available. The patient had no symptoms prior to SARS-CoV-2 infection and had PASC symptoms for five months after SARS-CoV-2 infection. The patient died of palliative sedation and his brain was submitted to the Netherlands Brain Bank (NHB; Amsterdam, the Netherlands).

### Ethics

All studies were approved by the Medical Research Ethics Committee of both hospitals and accepted by the competent authority, the Central Committee on Research on Human Subjects (MUSCLE-PASC: NL78394.018.21, S3: NL73478.029.20, Amsterdam UMC COVID-19 biobank material via approved protocol 2020.182) and conducted according to the declaration of Helsinki. Written informed consent was obtained from each participant for the MUSCLE-PASC and S3 study. The Amsterdam University Medical Centers (UMC) COVID-19 biobank prospectively included patients in the Covid-biobank if they were admitted to the Amsterdam UMC with COVID-19 and had provided written informed consent or had not used the opt-out form. Informed consent was obtained for both patients of whom brain tissue was analysed.

### Metabolomics

PBMC were isolated within 2 h after drawing blood, using standard procedures. 500,000 PBMC were pelleted and subjected to analyses. Metabolomics was performed as previously described, with minor adjustments.^16^ A 75 μL mixture of the following internal standards in water was added to each sample: adenosine-^15^N_5_-monophosphate (100 μM), adenosine-^15^N_5_-triphosphate (1 mM), D_4_-alanine (100 μM), D_7_-arginine (100 μM), D_3_-aspartic acid (100 μM), D_3_-carnitine (100 μM), D_4_-citric acid (100 μM), ^13^C_1_-citrulline (100 μM), ^13^C_6_-fructose-1,6-diphosphate (100 μM), guanosine-^15^N_5_-monophosphate (100 μM), guanosine-^15^N_5_-triphosphate (1 mM), ^13^C_6_-glucose (1 mM), ^13^C_6_-glucose-6-phosphate (100 μM), D_3_-glutamic acid (100 μM), D_5_-glutamine (100 μM), ^13^C_6_-isoleucine (100 μM), D_3_-leucine (100 μM), D_4_-lysine (100 μM), D_3_-methionine (100 μM), D_6_-ornithine (100 μM), D_5_-phenylalanine (100 μM), D_7_-proline (100 μM), ^13^C_3_-pyruvate (100 μM), D_3_-serine (100 μM), D_5_-tryptophan (100 μM), D_4_-tyrosine (100 μM), D_8_-valine (100 μM). Subsequently, 425 μL water, 500 μL methanol and 1 mL chloroform were added to the same 2 mL tube before thorough mixing and centrifugation for 10 min at 14,000 rpm in an Eppendorf centrifuge. The top layer, containing the polar phase, was transferred to a new 1.5 mL tube and dried using a vacuum concentrator at 60 °C. Dried samples were reconstituted in 100 μL methanol/water (6/4; v/v). Metabolites were analyzed using a Waters Acquity ultra-high-performance liquid chromatography system coupled to a Bruker Impact II™ Ultra-High Resolution Qq-Time-Of-Flight mass spectrometer. Samples were kept at 12 °C during analysis and 5 μL of each sample was injected. Chromatographic separation was achieved using a Merck Millipore SeQuant ZIC-cHILIC column (PEEK 100 × 2.1 mm, 3 μm particle size). Column temperature was held at 30 °C. Mobile phase consisted of (A) 1:9 acetonitrile:water and (B) 9:1 acetonitrile:water, both containing 5 mM ammonium acetate. Using a flow rate of 0.25 mL/min, the LC gradient consisted of: 100% B for 0–2 min, ramp to 0% B at 28 min, 0% B for 28–30 min, ramp to 100% B at 31 min, 100% B for 31–35 min. MS data were acquired using negative and positive ionization in full scan mode over the range of *m*/*z* 50–1200. Data were analyzed using Bruker TASQ software version 2022. All reported metabolite intensities were normalized to internal standards with comparable retention times and response in the MS. Metabolite identification has been based on a combination of accurate mass (relative) retention times and fragmentation spectra, compared to the analysis of a library of standards.

### Seahorse

Key parameters of mitochondrial function of PBMC from donors were measured with the Agilent Seahorse XF Cell Mito Stress Test on a XFe96 Seahorse (Seahorse Biosciences) as optimized for PBMC and reported.^17^ In short, frozen PBMC were thawed and counted (Coulter cell counter). 225 × 10^3^ cells per well (8 replicates) were plated on a Poly-D lysine (Sigma, use 50 μg/mL, 25 μl/well) coated plate in 50 μl of XF media pH 7.4 (RPMI-1640 Medium With l-glutamine, without glucose and sodium bicarbonate; R1383 van Sigma–Aldrich).

For background corrections (n = 16) wells were filled with XF medium only. After seeding, the plate was centrifuged for 5 min at 200 g at RT with acceleration 1 and brake 0 and an extra 130 μl of XF medium was added. Cells were placed in a 37 °C incubator without CO_2_ and measured within 45 min after centrifugation. The XF assay protocol started with 3 cycles of 5 min (mix 2 min, measure 3 min) for basal respiration and after every injection this measurement of 3 cycles was repeated (twelve cycles in total). Compounds used for the Mito stress test were: oligomycin (1.5 μM), FCCP (1.25 μM) and a mixture of rotenone (1.25 μM) plus antimycin A (2.5 μM).

We have provided both the raw data in the supplemental data (Supplemental Figure S2) and, for comparison, the normalized data using the formula: x normalized = (x – x minimum)/(x maximum – x minimum). Data was normalized at the value just before adding oligomycin.

### Other procedures

#### Immunohistochemistry

PBMC were cytospinned, air-dried and immunostained. The stain procedures as well as the analyses of the kynurenine metabolites in serum and plasma have been described in detail,^6^ in which the Supplement and Supplemental Figure S1 provides further characterization of the anti-IDO-2 antibody. Supplemental Table S3 provides an overview of applied antibodies. For comparative analyses cytospins were immunostained in parallel. During our study we noticed that immunostaining shortly after air-drying of the cytospins yielded better IHC data.

#### Quantitation of tryptophan, kynurenine and metabolites

A mix of stable isotope-labeled internal standards was added to 50 μL plasma or CSF. Samples were deproteinized with acetonitrile, dried under nitrogen and reconstituted in 100 μL acetonitrile. 10 μL extract was injected in UPLC-MS/MS system, comprising an Acquity XEVO TQ-XS system (Waters, Milford, Massachusetts, USA) operated in positive ESI mode using MRM's for preselected analytes and overall run time of 6 min.

The ARH antagonist (Alternative Names: IK-175; KYN-175) was kept as 100 mM stock in DMSO at 4 °C, and diluted in medium to 0.5 μM and 5 μM for incubation with 50,000 PBMC in 200 μL in a 96-well plate. 0.005% DMSO was used as vehicle control. PBMC were maintained in IMDM, with 10% FCS, 0.05 mg/mL gentamycin and 0.00036% (v/v) β-mercaptoethanol.

### Statistical analyses

#### General

Distributions were assessed with histograms and Shapiro–Wilk tests. Categorical values are noted in absolute numbers with percentages in the brackets. Parametric quantitative variables are presented as means of standard deviation (SD), and nonparametric quantitative variables are presented as median and interquartile ranges (IQR; 25th and 75th percentiles). Measurements below the limit of quantification were imputed as 1/10 of the limit of quantification.

Categorical data were analyzed using Chi-square or Fisher exact test. Continuous parametric data were analyzed using a t-test or analysis of variance. Continuous parametric post-hoc tests were done with Tukey HSD. Continuous non-parametric data were analyzed using the Kruskal–Wallis H test. Non-parametric post-hoc testing was performed using the pairwise Kruskal–Wallis test with Benjamini-Hochberg (BH) correction. p values less than 0.05 defined significance. All data were analyzed using R studio built under R version 4.0.3 (R Core Team 2013, Vienna, Austria).

#### Metabolomics

Identified metabolites were classified according to the Human Metabolome Database (HMDB). To identify discriminatory metabolites and their importance for the difference between the groups, the statistical analysis and visualization of the acquired data were done in a R environment using the ggplot2, ropls and mixOmics packages.^18^, ^19^, ^20^ From the orthogonal partial least squares regression ((O)PLS-DA) the Variable Importance Projection (VIP) scores were used to estimate the importance of each metabolite in the projection used in the PLS-model. A metabolite with a VIP-score equal or greater than 1 was considered important in the given PLS-model. A t-test was performed to assess significant differences in metabolite levels between the combinations of two groups. The HOLM-correction was used to adjust p-values for multiple testing. p-values lower than 0.05 were considered significant. Metabolome data was visualized using volcano plots with log2 fold changes. Correlations between the identified metabolites were performed on the log2-transformed data using Pearson's correlation and Spearmans rank correlation coefficient for non-linear relationships.

As an exploratory analysis, sparse Partial Least Squares—Discriminant Analysis (sPLS-DA; R package v. 3.6.1 and mixOmics v6.10.9; applied functions: *tune.splsda* and *splsda* was conducted between the clinical outcomes (Basal OCR, Maximal Respiration OCR, Spare Respiratory Capacity) and the Seahorse Metabolite dataset. *splsda* is a supervised, multivariate modelling strategy that selects the most discriminative features or variables in a dataset to classify samples or discriminate between groups of interest. The optimal number of features to be selected for a model in order to achieve the best performance, is determined via a tuning algorithm.^21^ *tune.splsda* derives the optimum number of components that result in the best overall error rate. For these analyses the respiratory outcomes were split into quartiles (Q1-4), after which models including all four groups and only Q1 and Q4 (extreme analyses) were derived. The process is based on one-sided t-tests, and checks whether the model performance improves when adding a component.^18^

## Results

### General characteristics of participants and analysis of tryptophan, kynurenine, and downstream catabolites

**Fig. 1:**
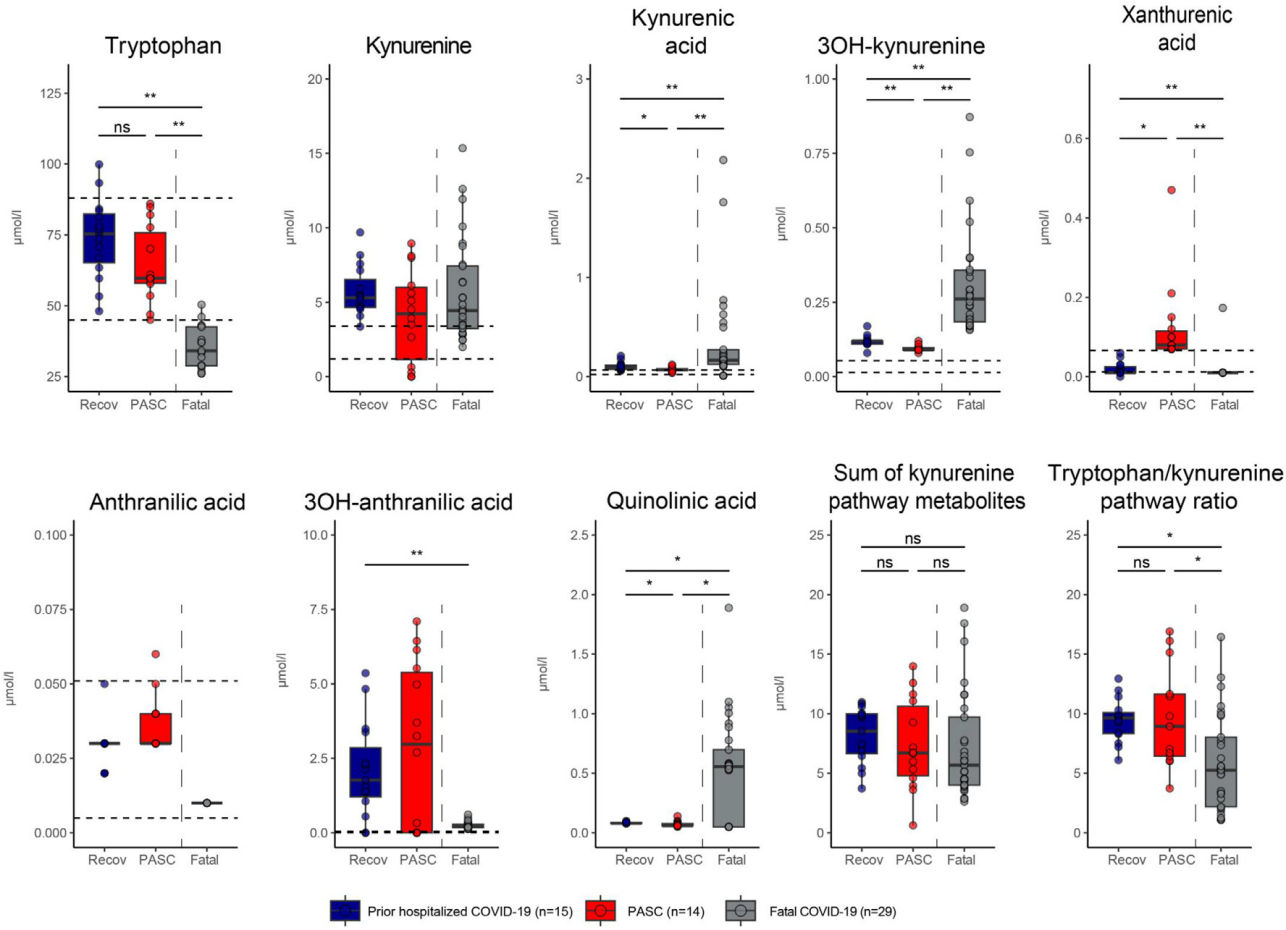
Kynurenine pathway metabolites in plasma from PASC patients, and recovered but prior hospitalized COVID-19 patients. For comparison we included values of plasma from patients with fatal/severe COVID-19 (reference^6^; these separate data have not been published before). Statistical analysis of parametric variables were performed using analysis of variance and post hoc tests with Tukey HSD. Non-parametric data were analysed using the Kruskal–Wallis H test with post hoc pairwise Kruskal–Wallis test with Benjamini-Hochberg correction. The area between the dotted lines represent the normal reference values as determined in reference 22 for plasma and reference 23 for serum. Metabolites in the fatal/severe group had several measurements at the lower limit of quantification for quinolinic acid, 3OH-anthranilic acid, kynurenic acid and xanthurenic acid, which were set at 1/10 of the lower limit of quantification. ns: not significant, ∗: p-value <0.05, ∗∗: p-value <0.001. Recov: Prior hospitalized COVID-19 patients PASC: Post-acute sequelae of COVID-19.

EDTA plasma samples for analysis of kynurenine and its downstream metabolites were obtained 134–498 days after infection from fifteen PASC patients, one of whom was excluded retrospectively from the analyses because proof of a SARS-CoV-2 infection was missing. None of these patients were admitted to the hospital during SARS-CoV-2 infection and all were in good health (no medical of psychiatric history) prior to SARS-CoV-2 infection. For comparison, we included EDTA plasma samples from 15 hospitalized COVID-19 patients who were sampled 60–208 days after infection and had no or mild remaining symptoms only. The concentrations of kynurenine and its downstream metabolites in plasma are shown in Fig. 1, in which we also included those in EDTA plasma from patients with fatal/severe COVID-19 analyzed earlier.^6^ In parallel, we also analysed serum samples (plasma samples were not available), obtained 62–154 days after infection from 12 non-hospitalized COVID-19 patients, all healthcare workers who were routinely measured for seroconversion since the beginning of the COVID-19 pandemic. There was no follow-up on remaining symptoms for these patients. For a complete group overview of all study participants see Table 1, and of all kynurenine analyses see Supplemental Table S4. All groups of SARS-CoV-2-infected individuals had metabolites of the kynurenine pathway present systemically, markedly contrasting with reference values.^22^^,^^23^ There were, however, marked differences between the groups for specific metabolites. In fatal/severe COVID-19 we found the highest levels for kynurenine-metabolites (3OH-kynurenine, quinolinic acid, kynurenic acid), whereas levels of tryptophan and 3OH-anthranilic acid were lowest in comparison to the other groups. In PASC patients levels of xanthurenic acid were highest, and, although not significant, the median 3OH-anthranilic acid level was highest in PASC patients (up to 106-fold higher than the mean reference value; even though 5 out of 14 patients had no detectable 3OH-anthranilic acid) as opposed to 7.5-fold higher in fatal/severe COVID-19 (where all had detectable 3OH-anthranilic acid). Even in patients who were either hospitalized with no or only mild remaining symptoms, or were never hospitalized, increased median levels of 3OH-anthranilic acid were found (respectively, 63-fold higher than the mean reference value; with 2 out of 15 patients with no 3OH-anthranilic acid and 25-fold higher than the mean reference value (for serum); where all had detectable 3OH-anthranilic acid). It is worth noting that patients in all groups had enhanced systemic kynurenine and downstream metabolites, even though the actual infection was at least more than two months prior to blood sampling, and even a median 294 days for PASC patients.

**Table 1:** Characteristics of study participants of whom EDTA plasma or serum was analyzed for kynurenine metabolites.

	PASC	Non-hospitalized Covid-19	Hospitalized Covid-19	p-value	Fatal/severe
	n = 15	n = 12	n = 15		n = 12
Age (median [IQR])	49.00 [42.50, 54.00]	51.00 [39.25, 60.00]	61.00 [54.50, 65.00]	0.004	67.50 [64.25, 74.25]
Gender = Male (%)	3 (20.0)	3 (25.0)	9 (60.0)	0.048	10 (83.3)
PCR positive (%)				<0.001	
No	1 (6.7)	0 (0.0)	0 (0.0)		0 (0.0)
PCR	12 (80.0)	4 (33.3)	15 (100.0)		12 (100.0)
Wantai	2 (13.3)	8 (66.7)	0 (0.0)		0 (0.0)
Days after infection sampled (median [IQR])	294.00 [237.00, 426.50]	91.50 [90.25, 95.75]	108.00 [95.50, 127.00]	<0.001	
Recovered = Yes (%)	0 (0.0)	12 (100)	7 (46.7)		0 (0.0)^a^
Infection before vaccination = Yes (%)	15 (100.0)	12 (100.0)	15 (100.0)	>0.99	12 (100.0)
Residual symptoms = Yes (%)	15 (100.0)	0 (0)	8 (53.3)		
Days of hospitalization (median [IQR])	0 (0)	0 (0)	9.00 [6.00, 11.50]		12.5 [9.75, 16.00]
ICU admission = Yes (%)	0 (0)	0 (0)	5 (33.3)		12 (100.0)
Intubation = Yes (%)	0 (0)	0 (0)	2 (14.3)		12 (100.0)

The fatal/severe patients have been described in reference 6, and were just added for comparison and not included in the statistical analyses. n.k.: not known.

Parametric quantitative variables are presented as means of standard deviation (SD), and nonparametric quantitative variables are presented as median and interquartile ranges (IQR; 25th and 75th percentiles).

Categorical data were analyzed using a chi-square test. Continuous parametric data were analyzed using analysis of variance. Continuous non-parametric data were analyzed using the Kruskal–Wallis H test.

a 10 deaths, 1 unknown after transfer, 1 not recovered after 3 months.

### Active IDO2 expression driven by the AHR

Next, we aimed to clarify whether this degradation of tryptophan was due to IDO2 and/or its isoform IDO1. From our previous study^6^ we knew that in fatal COVID-19 various blood cells express IDO2 and thus we analyzed cytospins from purified PBMC, containing monocytes and lymphocytes from non-hospitalized PASC patients (n = 9) and fully recovered SARS-CoV-2-infected patients (n = 14; overview in Table 2) from the MUSCLE-PASC study. PASC patients were sampled a median of 513 [IQR 420–599] days after initial infection and had fasted for a minimum of 2 h before drawing blood.

**Table 2:** Characteristics of patients and participants of whom peripheral blood monocytes were analyzed by immunohistochemistry, metabolomics and ‘Seahorse’.

	Infected and recovered	PASC	p-value
	n = 14	n = 9	
Age, median (IQR)	31.50 [29.00, 44.25]	43.00 [28.00, 50.00]	0.131
Sex, Male (%)	8 (57.1)	2 (22.2)	0.253
Positive PCR (%)	14 (100.0)	9 (100.0)	0.999
Days after infection sampled, median (IQR)	254.50 [78.50, 556.75]	513.00 [434.00, 599.00]	0.166
Recovered after SARS-CoV-2 infection (%)	14 (100.0)	0 (0.0)	<0.001
SARS-CoV-2 infection before vaccination, (%)	7 (50.0)	9 (100.0)	<0.001
Working hours as compared to before SARS-CoV-2 infection (%)	100.00 [100.00, 100.00]	37.50 [15.00, 41.67]	<0.001

Parametric quantitative variables are presented as means of standard deviation (SD), and nonparametric quantitative variables are presented as median and interquartile ranges (IQR; 25th and 75th percentiles).

Categorical data were analyzed using a Fishers exact test. Continuous parametric data were analyzed using a t-test. Continuous non-parametric data were analyzed using the Mann–Whitney U test.

**Fig. 2:**
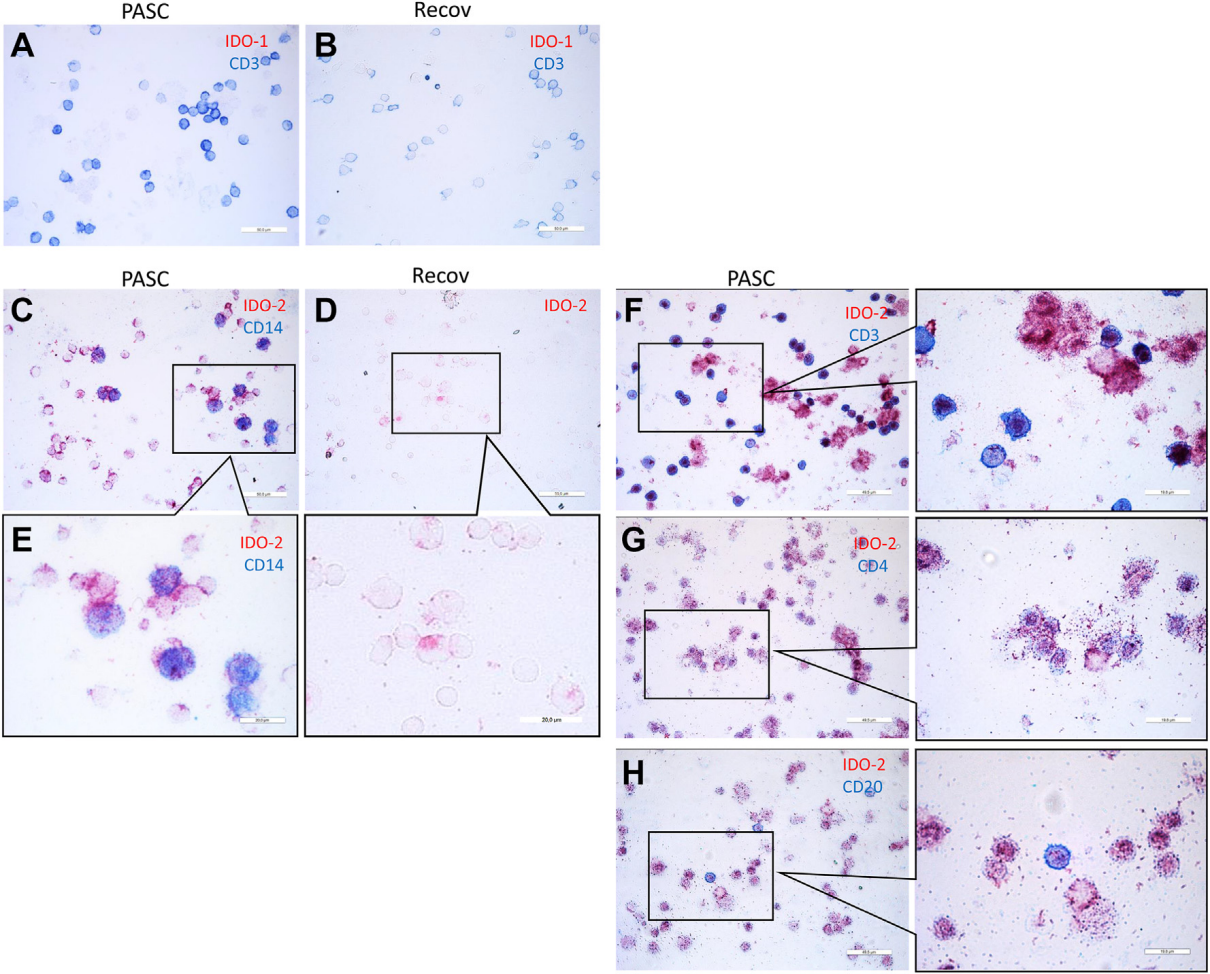

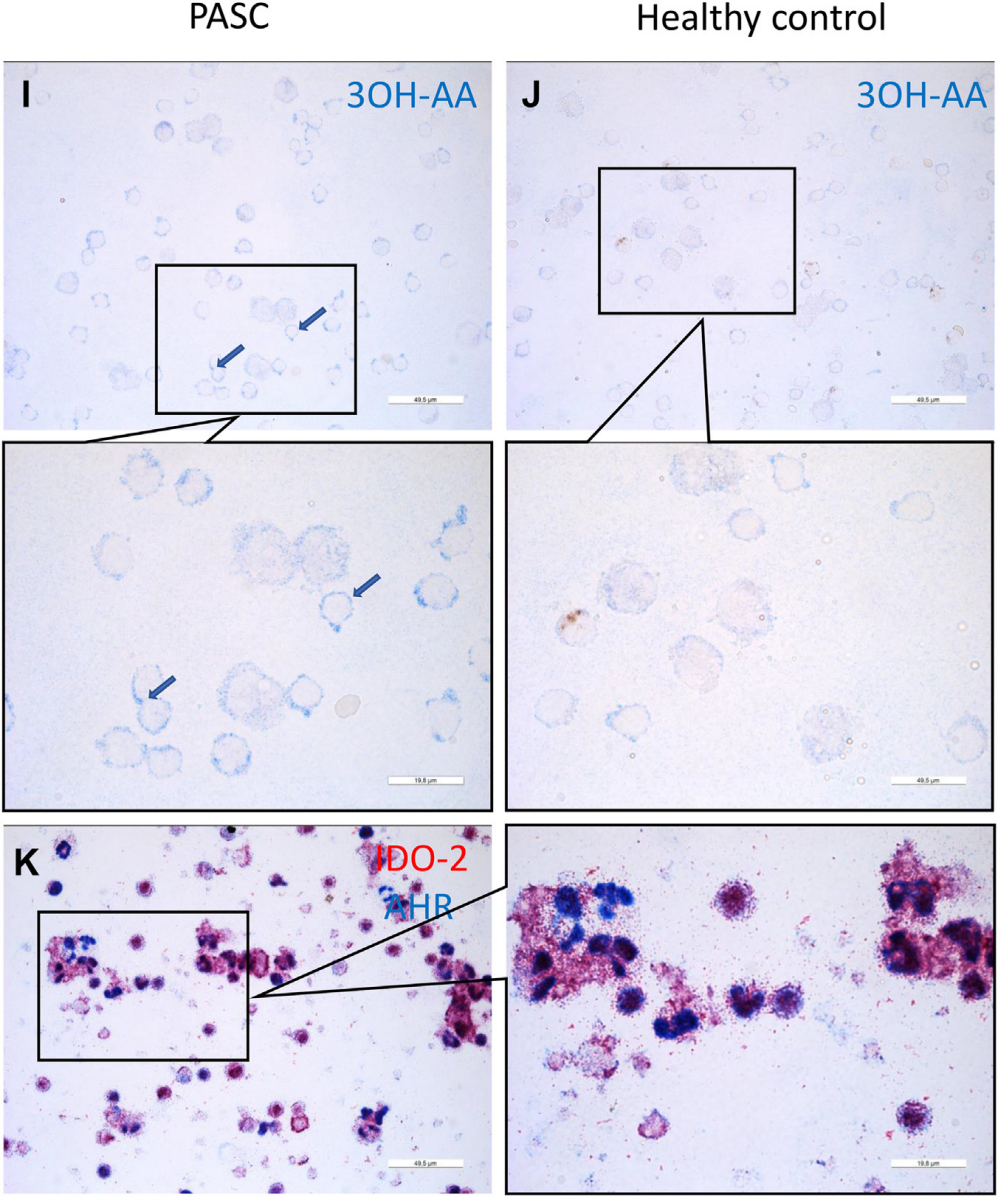
Immunohistochemical analyses of peripheral blood mononuclear cells (PBMC) from PASC patients and SARS-CoV-2-infected participants fully recovered (Recov). Cytospins were prepared directly after isolation of PBMC. Absent IDO1 stain in PASC and Recov (A and B; a positive control was done separately: not shown). IDO2 stain in PASC and recover (C and D). IDO2-expressing cells are predominantly monocytes, CD4 lymphocytes and B lymphocytes (C, E–H). 3OH-anthranilic acid was shown in PBMC from PASC patients and not in those from healthy individuals (I, J). Nuclear localization of AHR in IDO2-expressing PBMC from PASC patients (K).

IDO1 was absent in cytospins from PASC patients (n = 9), whereas IDO2 was abundantly present (Fig. 2A and C). In response to a reviewer's comment and due to fading of the immunostain, we were able to establish the number of IDO-2-positive cells in 4 PASC patients only, which were 100%, 100%, 100% and 93%. In cytospins from the fully recovered patients (n = 10), IDO1 stain was also negative and the IDO2 stain showed some immunoreactivity (Fig. 2B and D), but was markedly lower than that for PASC patients. IDO2 in PASC and the fully recovered patients was found in monocytes (CD14+) and lymphocytes (CD3+), predominantly CD4+ and CD20+ lymphocytes (Fig. 2C, E, F, G and H). 3OH-anthranilic acid stained near the cell membrane in line with the enhanced levels of 3OH-anthranilic acid in plasma from PASC patients (Fig. 2I; cf. Fig. 2J). Similar to our findings in fatal COVID-19, we found that the AHR was localized in the nucleus of IDO2-positive cells (Fig. 2K), in line with the concept that AHR is driving IDO2 expression.

**Fig. 3:**
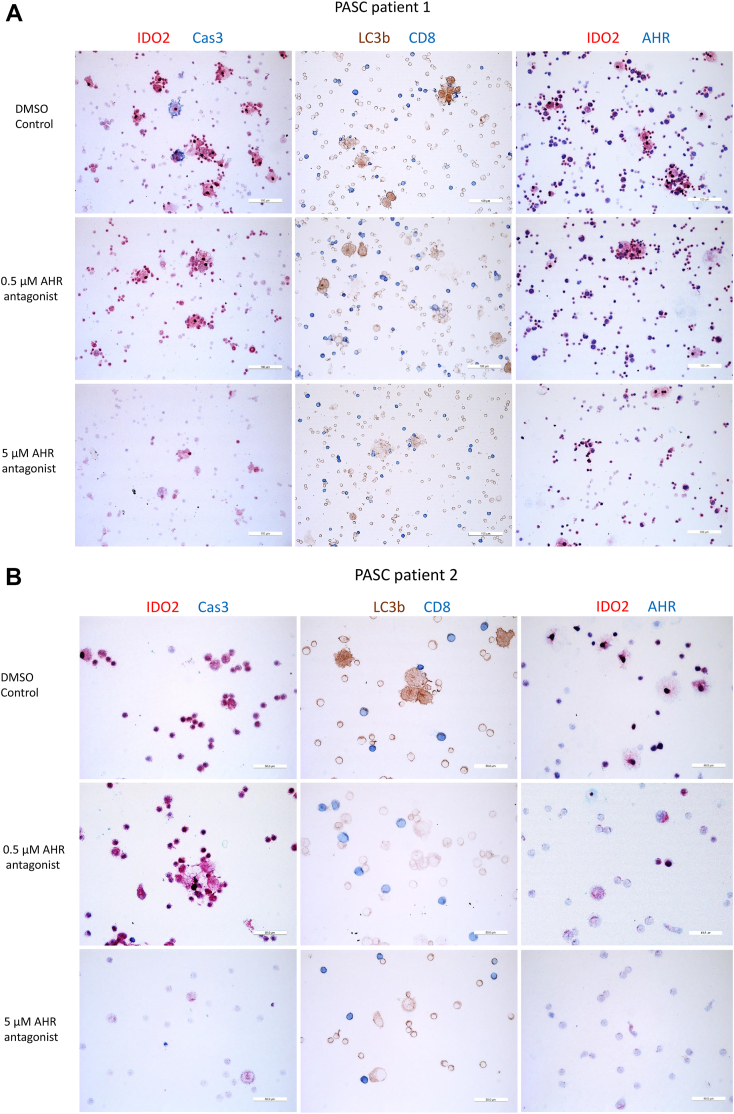
Dose-dependent inhibition of IDO2 expression, autophagy and apoptosis by an aryl hydrocarbon receptor (AHR) antagonist. Peripheral blood mononuclear cells were cultured for 8 days in the presence of the AHR antagonist at the indicated concentration or with vehicle (DMSO) only. Immunostains for two PASC patients (A and B) are shown. Cells were evaluated for viability daily by microscopy. Cas3 is activated caspase 3, which is a marker of apoptosis. LC3b is a marker of autophagy.

To substantiate that AHR drives IDO2 expression, we cultured PBMC from 5 PASC patients for 8 days, in the presence of 0.5 or 5 μM of the AHR antagonist or with vehicle (0.005% DMSO) only. IDO2 expression was inhibited in a dose-dependent manner and almost eradicated at 5 μM (Fig. 3A and B). IDO2-expressing cells displayed the marker for autophagy (L3CB), but not or minimally, that of apoptosis (cleaved-caspase 3). Incubation with the AHR antagonist inhibited autophagy and apoptosis. The AHR remained in the nucleus despite treatment with the AHR antagonist.

### Aberrant metabolism and reduced mitochondrial functioning in PBMC from PASC patients

**Fig. 4:**
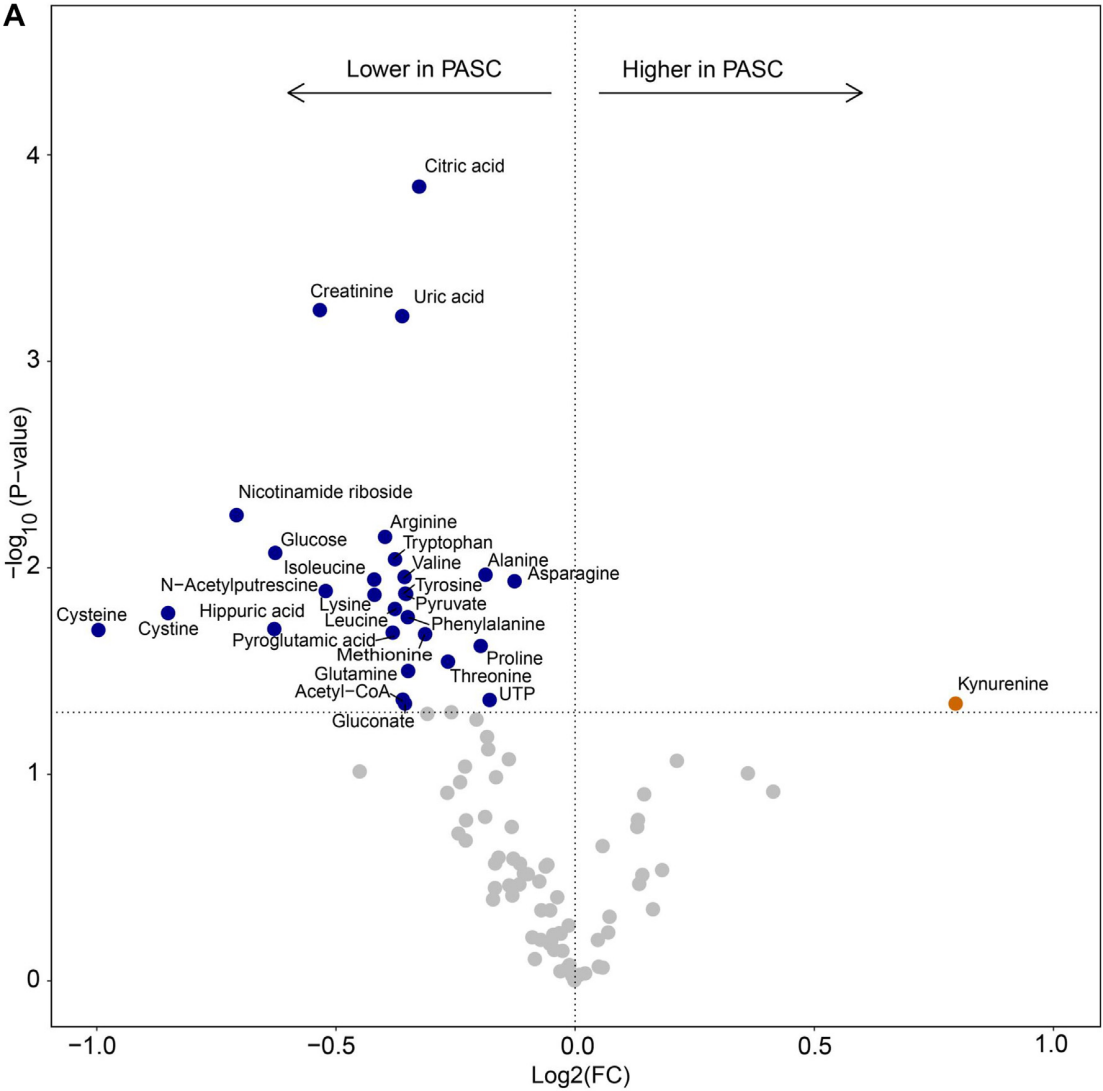

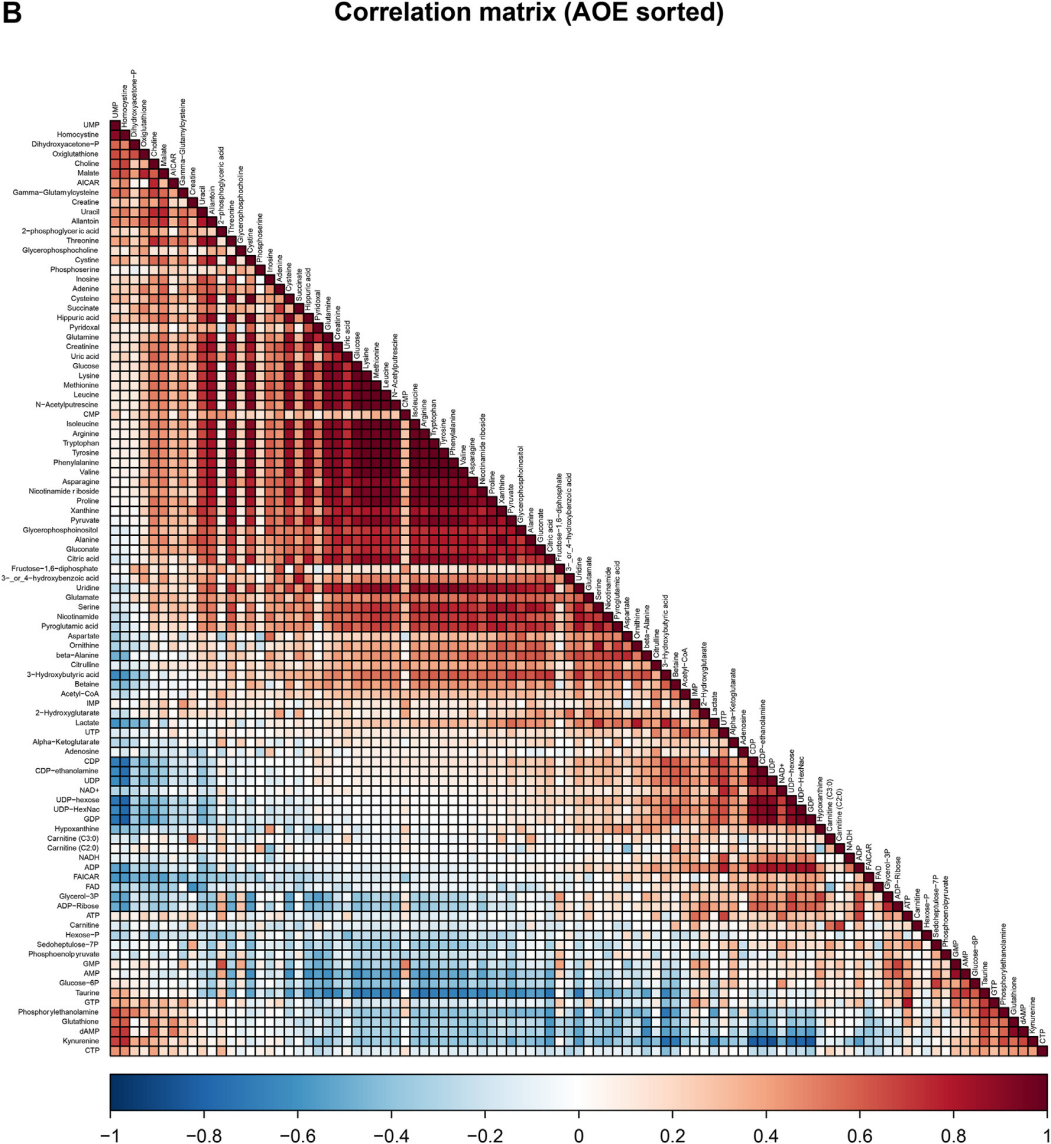
Metabolomics data for peripheral blood mononuclear cells from PASC patients compared to that from SARS-CoV-2-infected patients who recovered in full (recover). Volcano plot with colored symbols for significantly altered metabolites (A). Correlation plot for metabolites (B). As these analyses were explorative and with a relatively low number of samples, no correction for multiple testing was performed.

As most PBMC from PASC patients expressed IDO2, we next analyzed 500,000 PBMC from 9 PASC patients and 14 fully recovered patients from the MUSCLE-PASC study by metabolomics for 219 metabolites covering most of the central carbon metabolic pathways, including glycolysis, TCA cycle, amino acids, pentose phosphate pathway, and NAD metabolism.^16^ The cellular composition of the PBMC population did not differ between both groups. In PASC patients we found 82.54 ± 3.02% (mean ± S.D.) lymphocytes, 12.91 ± 1.77% monocytes, and 4.55 ± 2.30% granulocytes, whereas for recovered non-hospitalized patients these numbers were 83.90 ± 3.03% lymphocytes, 12.53 ± 2.46% monocytes, and 3.57 ± 1.63% granulocytes. Retrospectively, we excluded metabolomics data from one PASC patient and one recovered patient as they were outliers. We identified 97 metabolites and in order to normalize metabolomics data we log-transformed data without further normalization. Amongst anthranilic acid, kynurenic acid, kynurenine, quinolinic acid and tryptophan in the metabolic analysis, kynurenine was enhanced and tryptophan was reduced in PBMC from PASC patients compared to those from the fully recovered patients (Fig. 4A). This confirms that the observed IDO2 expression in PBMC from PASC patients is metabolically active, more than in those from recovered patients. Besides tryptophan, 14 amino acids were present at reduced levels in PBMC from PASC patients. Finally, the reduced amounts of pyruvate, acetyl-CoA, citric acid and nicotinamide riboside may relate to an altered activity of the Krebs cycle and of mitochondria in general. The correlation plot (Fig. 4B) shows that these significant differentiating metabolites correlate, indicative of a genuinely affected metabolism in IDO2-expressing cells. It is also worth noticing that several nucleoside phosphates correlated with each other, and kynurenine correlated with anti-oxidant compounds (lower left corner of the correlation plot).

**Fig. 5:**
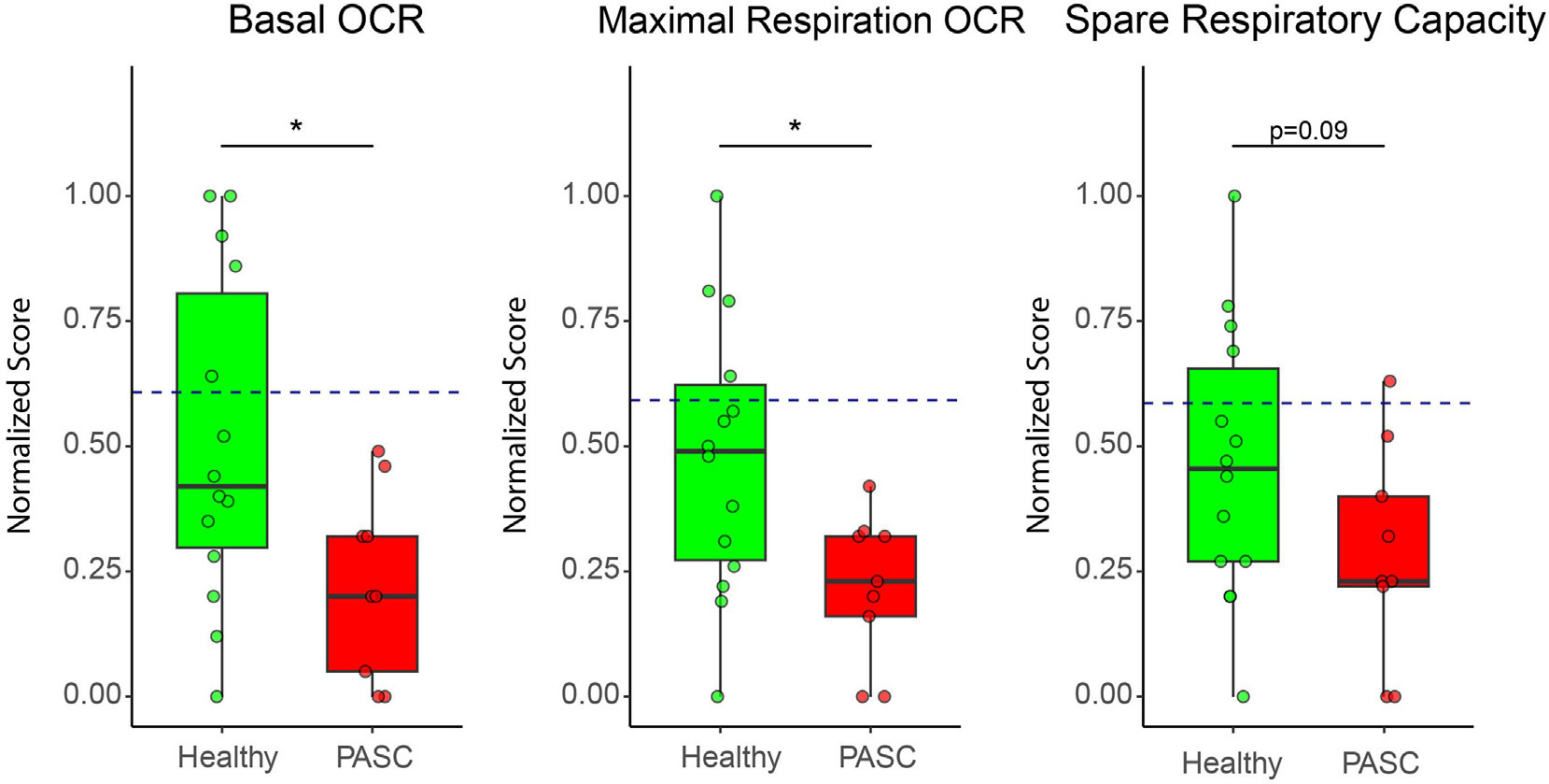
Normalized respirometry data showing the oxygen consumption rate and spare respiratory capacity for peripheral blood mononuclear cells from PASC patients and participants fully recovered from a SARS-CoV-2 infection without hospitalization (Healthy). The blue dashed line represents the mean respiration value of participants who had not yet experienced a SARS-CoV-2 infection (non-COVID-19). All values were normally distributed and analysed with a t-test, which was followed with Benjamini-Hochberg (BH) correction. ∗: p-value<0.05.

The enhanced levels of 3OH-anthranilic acid, which is an intermediate in the synthesis of NAD, and the reduced levels of nicotinamide riboside may relate to an aberrant mitochondrial activity in IDO2 expressing cells. Therefore, we performed mitochondrial stress tests with PBMC (9 from PASC and 14 from recovered patients) using Agilent Seahorse XF respirometry. As a control we also ran the analyses with PBMC obtained from 5 healthy individuals, who claimed that they had not been infected by SARS-CoV-2 before PBMC collection. The traces of the separate runs are provided as Supplemental Figure S2. After normalization we could compare the data of the various runs (Fig. 5). Basal and maximal oxygen consumption rates (OCR) were reduced in PBMC from PASC patients compared to both recovered patients and healthy individuals. The OCR did not differ between PBMC from recovered patients and from healthy controls. Compared to PBMC from healthy controls, the spare respiratory capacity (SRC) was reduced in PBMC from recovered patients as well as from PASC patients. We did not find a correlation between levels of kynurenine and of nicotinamide riboside with the OCR and SRC values (Supplemental Figure S3). Next, we performed an exploratory sparse Partial Least Squares—Discriminant Analysis (sPLS-DA),^20^ which is a supervised, multivariate modelling strategy, to select the most discriminative variables in the metabolite dataset to discriminate between high and low Seahorse data. The basal and maximal OCR and SRC values, split into quartiles, did not show discriminative power. Only when the most divergent quartiles (Q1 *versus* Q4) were analysed, the metabolites citric acid, glutamine, cysteine, arginine and cystine, were the most important discriminators between high and low OCR and SRC values.

### IDO2 stain in brain tissue from PASC patients

While this manuscript was being revised, we obtained autopsy brain material from two PASC patients, which allowed us to determine whether IDO2 expression was present and whether viral antigen was detectable. There was abundant IDO2 expression in brain tissue, which was spatially related to 3OH-anthranilic acid and quinolinic acid, and with nuclear, i.e. transcriptionally active AHR (Supplemental Figures S4 and S5). In both brains, and in association with IDO2 expression, abundant autophagy and limited apoptosis was seen, although marked numbers of apoptotic cells were seen under the ependymal cells. Unexpectedly, we also found IDO1 stain (not shown) overlapping with IDO2 stain in the brain, although there were areas with IDO2 stain only. Both brains were immunostained for the N-protein of SARS-CoV-2, as described previously,^24^ and were negative.

## Discussion

With this study we aimed to clarify whether IDO2 is expressed and active in PASC, and may contribute to PASC symptoms and pathophysiology. We showed that monocytes and lymphocytes of PASC patients collected 294 (median) days after the SARS-CoV-2 infection indeed displayed IDO2 activity, which was paralleled by enhanced levels of kynurenine metabolites in the circulation, particularly xanthurenic acid and for some patients also highly enhanced levels of 3OH-anthranilic acid. The IDO2-expressing PBMC had high kynurenine and low tryptophan levels and an aberrant cellular metabolism reflected predominantly by autophagy and a reduced mitochondrial functioning. IDO2 expression and activity was halted by an AHR antagonist. Immunohistochemical analysis of brain tissue from two PASC patients showed a pronounced IDO2 expression, IDO activity and predominant autophagy with some areas with apoptotic cells.

In our previous study^6^ we have extensively validated the specificity of the used IDO2 antibody. Here we studied two cohorts of PASC patients; in one we confirmed IDO activity in plasma, in the other expression in PBMC of IDO2 and excluding that of IDO1. In addition, we showed that IDO2-expressing PBMC from PASC patients had reduced cellular levels of tryptophan and enhanced levels of kynurenine. Finally, we showed that IDO2 expression was reduced upon treatment of PASC PBMC with the AHR antagonist. Together this can be taken as proof that in PASC there is IDO2 expression and activity.

IDO2 expression and activity is manifest in fatal COVID-19, PASC and even in SARS-CoV-2-infected patients who fully recovered. This suggests that IDO2 expression is triggered by SARS-CoV-2 infection, in line with an earlier report on AHR activation for murine coronavirus infection in mice,^14^ and in humans for infection with coronavirus, among which SARS-CoV-2.^15^ The difference between these studies and our current study is that we assessed IDO2 expression and activity at far later time points after infection. Although we failed to detect viral proteins in IDO2-expressing brain tissue from PASC patients, we cannot rule out that in the PASC cohort there is still virus present in other tissues, as has been claimed in several reports.^25^^,^^26^ IDO2 expression and activity in fatal/severe COVID-19, however, was in the absence of viral particles in tissue using two different antibodies against SARS-CoV-2,^24^ indicating that the presence of virus is not required for IDO2 expression at that stage.

The activity of the kynurenine-AHR-IDO2 axis is controlled by the AHR repressor (AHRR), which can interfere with AHR-induced transcription.^27^ The expression and activity of AHRR is subject to regulation. In SARS-CoV-2-infected individuals,^28^ obese patients^29^ and smoking individuals^30^ the AHRR expression and/or activity is attenuated. Therefore, we consider it more likely that the IDO2 expression results from a failure to antagonize AHR-induced transcription of IDO2. The resulting IDO2 expression generates kynurenine which again activates the AHR, leading to further IDO2 expression, and thus to a positive feedback loop. The continued expression of IDO2 in the various SARS-CoV-2-infected patients is in line herewith.

Although the involvement of the kynurenine pathway in COVID-19 was recognized early,^31^ several recent publications indicated that systemic kynurenine is a marker for acute COVID-19 and PASC, and even a prognostic marker for fatal COVID-19.^32^, ^33^, ^34^ Whereas we did find that plasma/serum kynurenine was higher in SARS-CoV-2-infected individuals compared to reference values, the kynurenine levels did not differentiate between the various study cohorts. This in itself is not that surprising as the generation of kynurenine is the rate-limiting step in the kynurenine pathway, and thus generated kynurenine is rapidly metabolized further. In addition, we noticed that taking tryptophan and all measured kynurenine metabolites in plasma in consideration (Fig. 1 and Supplemental Table S4), there is similar IDO2 activity between PASC, fatal/severe COVID-19 and Recov, although in the fatal/severe group some patients had much higher values (Fig. 1). It should be noted that the timeline of sampling varied between the patient groups, with samples of the control groups being obtained at earlier time points after SARS-CoV-2 infection than those of the PASC patients. Furthermore, kynurenine and several of its downstream metabolites spread systemically and thus, in the absence of adequate AHRR activity, can induce IDO2 at sites throughout the body. This was illustrated for example by IDO2 expression in the ependymal cells and cells in the subventricular zone of the brain in fatal/severe COVID-19,^6^ which are in close contact with cerebrospinal fluid that contained kynurenine. Similar observations apply to the immunostains of brain tissue from the two PASC patients, and so the plasma levels of kynurenine and downstream metabolites likely are not produced by PBMC only. And finally, IDO2 and the enzymes catabolizing kynurenine are cytoplasmic. The plasma levels of kynurenine and its downstream metabolites therefore may not reflect the levels at the cellular level and are influenced by cellular release (active release and through cell lysis), uptake by cells, and clearance of these metabolites. Therefore, the plasma levels should be taken primarily as an indication of IDO activity. Nevertheless, the dominant kynurenine metabolites vary between the different patient groups. In plasma from fatal COVID-19 there is an abundance of 3OH-kynurenine, quinolinic acid and kynurenic acid as well as a reduced tryptophan level, whereas in that from PASC and SARS-CoV-2-infected individuals we found enhanced levels of 3OH-anthranilic acid, and in PASC only that of xanthurenic acid. The various kynurenine and related metabolites exert a range of biological and even opposing activities, ranging from neuro- and cytotoxicity, but also neuro-protection, to protection against reactive oxygen species, and modulating NAD synthesis. It remains to be determined whether these differences relate to the underlying pathology due to SARS-CoV-2 infection. For example, in fatal/severe COVID-19, an abundance in the levels of neurotoxins, such as 3-hydroxykynurenine (3-HK) and quinolinic acid, was observed when compared to other groups. Despite a concurrent rise in the levels of the neuroprotective kynurenic acid, the overall balance appears to tip in favor of neurotoxicity, which could explain the high incidence of apoptotic cells observed during the severe course of the disease.^35^ In contrast, we found enhanced levels of xanthurenic acid in PASC patients, which has been reported to affect mitochondrial activity.^36^

The symptoms and features associated with PASC are diverse, ranging from fatigue, post-exertional malaise, cognitive problems, enhanced levels of cytokines and chemokines,^37^ and autoantibodies.^38^ As most PBMC expressed IDO2, we assessed respirometry and metabolomics on PBMC without further purification. The basal and maximal oxygen consumption rate in PBMC from PASC patients as a measure of mitochondrial functioning were reduced compared to that from recovered patients and controls who claimed not to be infected by SARS-CoV-2. In addition, the SRC was reduced in PBMC from PASC patients as compared to those controls, but not between PASC and recovered patients. These findings indicate that IDO2-expressing monocytes and lymphocytes from PASC patients display a reduced capacity to respond to stress conditions. The pathophysiological mechanisms leading to these aberrant mitochondrial activities are unclear, although xanthurenic acid may be involved.^36^ The sPLS-DA should be cautiously interpreted as due to the low number of required features (number of metabolites > number of samples), these models cannot be considered as very reliable and robust. The found discriminatory metabolites citric acid, glutamine, cysteine, arginine and cystine, should therefore be interpreted as indicative only. The combination of metabolites suggests that the variance in mitochondrial activity may relate to the extent of oxidative degradation of amino acids.

Besides low levels of tryptophan in PBMC from PASC patients, cellular levels of many amino acids were reduced. Whereas the reduced levels of tryptophan may lead to the observed autophagy, it is unclear why levels of the other amino acids are reduced. A possible explanation lies into the competition between kunurenine and multiple amino acids for transport by the large neutral amino acid transporter.^39^ Previously, we have shown that depletion of tryptophan in airway epithelial-like cells leads to the enhanced release of IL-6 and CXCL8, which was normalized by adding tryptophan.^40^ Similar exaggerated cytokine responses due to partial inhibition of protein synthesis have been well established for various cell types, among which monocytes and lymphocytes, and is referred to as superinduction.^41^, ^42^, ^43^ Therefore, the exaggerated cytokine (e.g., IL-6) and chemokine responses that are seen in PASC may relate to IDO2 activity.

Finally, several reports have implicated IDO2 activity in the generation of autoantibodies,^44^^,^^45^ another feature in PASC. Whether IDO2 activity indeed determines PASC pathophysiology may be addressed in clinical trials targeting the IDO2 pathway.

In this study we provided evidence for IDO2 activity in PASC patients. A particular strength of this study is that we had data on the health status of these patients before infection with SARS-CoV-2, allowing us to relate PASC symptoms to the infection. There are, however, a couple of limitations in the study. First, as our PASC study population was relatively small and the PASC diagnosis comprises a broad and heterogeneous panel of symptoms, an important potential limitation is to what extent the studied PASC population is representative of the PASC patients. For this study we included non-hospitalized patients, with a proven SARS-CoV-2 infection, who were healthy before infection and developed long-lasting PASC symptoms (post-exertional malaise, cognitive symptoms, fatigue). Non-hospitalization is important as hospitalization can induce various forms of fatigue, such as cachexia and sarcopenia.^5^ With that we met the WHO criterium: “PASC is defined as the continuation or development of new symptoms 3 months after the initial SARS-CoV-2 infection, with these symptoms lasting for at least 2 months with no other explanation”.^46^ By sticking to these strict inclusion criteria, and having patients in our cohort that vary with respect to intensity and specific PASC symptoms, we indeed believe that we have analyzed a representative and generalizable cohort of PASC patients. Confirmation in a larger population, also with hospitalized PASC patients, however, is required. Second, the reference values used in Table 2 are not determined in parallel to our analyses and thus are a potential source of bias. Having mentioned that, the tryptophan levels of the non-fatal patients were very similar to the reference values. Given the methodology of analyses and the chemical similarities of the kynurenine metabolites with tryptophan it is therefore likely that there may be a limited bias only. More importantly, the plasma samples from the PASC and recovered patients were run in parallel and those from fatal COVID-19 were run using the same method but at an earlier stage and thus can be compared reliably. Third, and related to the previous limitation, IDO2 expression in PBMC was determined qualitatively and not quantitatively. The primary reason was that quantifying IDO2 expression does not necessarily relate to IDO2 activity. Quantification of IDO2 stain would have required to immunostain all samples in parallel, which was logistically impossible. More importantly though, we expected that IDO2 in other tissues would also contribute to plasma levels of kynurenine and downstream metabolites. This is likely, now that we confirmed IDO2 stain in brain tissue from PASC patients. Another potential limitation is that, given the significant differences in ‘Age’, ‘Days after infection sampled', and ‘infection before vaccination’ between the groups (Table 1), for our analyses we did not adjust for these variables. This was not done as we looked for IDO activity and had no specific hypothesis that would be related to any of these variables, apart from of course SARS-CoV-2 infection. Furthermore, the hospitalized patients were a different group by design, as at the time young and otherwise healthy individual were not admitted. And, also the PASC and hospitalized groups belong to different disease entities, i.e. PASC *versus* COVID-19. Another potential limitation referring to the statistical analyses was that the metabolomics data were not corrected for multiple testing, which may have led to false positive signals. As many identified metabolites, however, correlated strongly and in an apparent meaningful manner, we consider our findings as genuine. Finally, our findings are predominantly for blood. The immunohistochemical analyses of brain tissue was from two PASC patients only, and although the diagnosis PASC was established in both patients, one suffered from comorbidities and for the other the medical history was not yet available, and therefore these findings are not conclusive. On the other hand the areas affected in brain tissue are similar to those in fatal/severe COVID-19, but with more autophagy than apoptosis in PASC compared to fatal/severe COVID-19. These preliminary findings urge further research into IDO2 expression and activity in brain tissue of PASC patients, into the presence of specific kynurenine metabolites and mitochondrial functioning of cells, and long-term effects into the functioning of various brain regions.

Whether IDO2 activity drives the pathology of PASC and even in fatal/severe COVID-19 needs to be established by inhibiting the IDO2-kynurenine axis. To the best of our knowledge there are currently no potent IDO2 inhibitors that can be applied in humans. Although the inhibitor of IDO1, the l-stereoisomer of 1-methyltryptophan (1-MT), or its d-stereoisomer have been claimed to inhibit IDO2, this is still disputed.^47^, ^48^, ^49^ Our studies with PBMC and the AHR antagonist suggest that the antagonist can attenuate IDO2 expression in PASC patients, likely to restore cell functioning. As the AHR is a ligand dependent transcription factor that can be driven by various ligands, it may be that some antagonist work better than others. From our *in vitro* studies it is clear that responses to the AHR antagonist differ between patients, and so the duration of treatment may differ between patients.

Symptoms of PASC are related to symptoms of patients with chronic fatigue syndrome, but although a link with IDO2 expression was proposed, be it as an enzymatically inactive enzyme,^50^^,^^51^ there is no proof of IDO2 expression in this heterogeneous patient group. Recent studies, however, found markedly increased levels of kynurenine and its downstream metabolites in relation to chronic fatigue syndrome.^52^^,^^53^ Therefore, our findings may possibly be relevant to patients with chronic fatigue syndrome.

Given the burden of PASC for patients as well as for society, our findings warrant a proof-of-concept study with AHR antagonist in PASC patients. There are a couple of pharmaceutical companies that have an AHR antagonist in their portfolio, which are nearing completion or completed phase 1 trials. These would be ideal candidates to try and inhibit IDO2 expression and activity in PASC patients.

## Contributors

LG, AD, TD and BSS performed experiments; LG, BA, RHH, MvW, FMV, PB and RL performed analyses and interpreted findings; LG, BA and RL accessed and verified all data and wrote the manuscript, BA, KM-K, JJS, AHAL, RCIW and MvV supervised the cohorts and selected patients; JWD supervised experiments and MB provided materials. RL designed and supervised the study. All authors had access to the original data, read and approved the final version of the manuscript. The Amsterdam UMC COVID-19 Biobank Study Group was responsible for the clinical care of COVID-19 patients and helped to collect, provide, and select plasma samples.

## Data sharing statement

There will be no public dataset, but any reasonable request for additional data will be honoured. Requests concerning the various cohorts will be forwarded to the principal investigators of those cohorts.

## Declaration of interests

The authors declare no competing interests.

## Appendix A Supplementary data

Supplementary Tables and Figures

3 Okt 2022 Amsterdam UMC Covid-19 Biobank investigators
