# Supplementary material for: Prolonged indoleamine 2,3-dioxygenase-2 activity and associated cellular stress in post-acute sequelae of SARS-CoV-2 infection
Source: eBioMedicine. 2023 Jul 26;94:104729. doi: 10.1016/j.ebiom.2023.104729 (PMC10406961; doi:10.1016/j.ebiom.2023.104729)
Supplement: Supplementary Tables and Figures [file mmc1.pptx]

## Slide 1
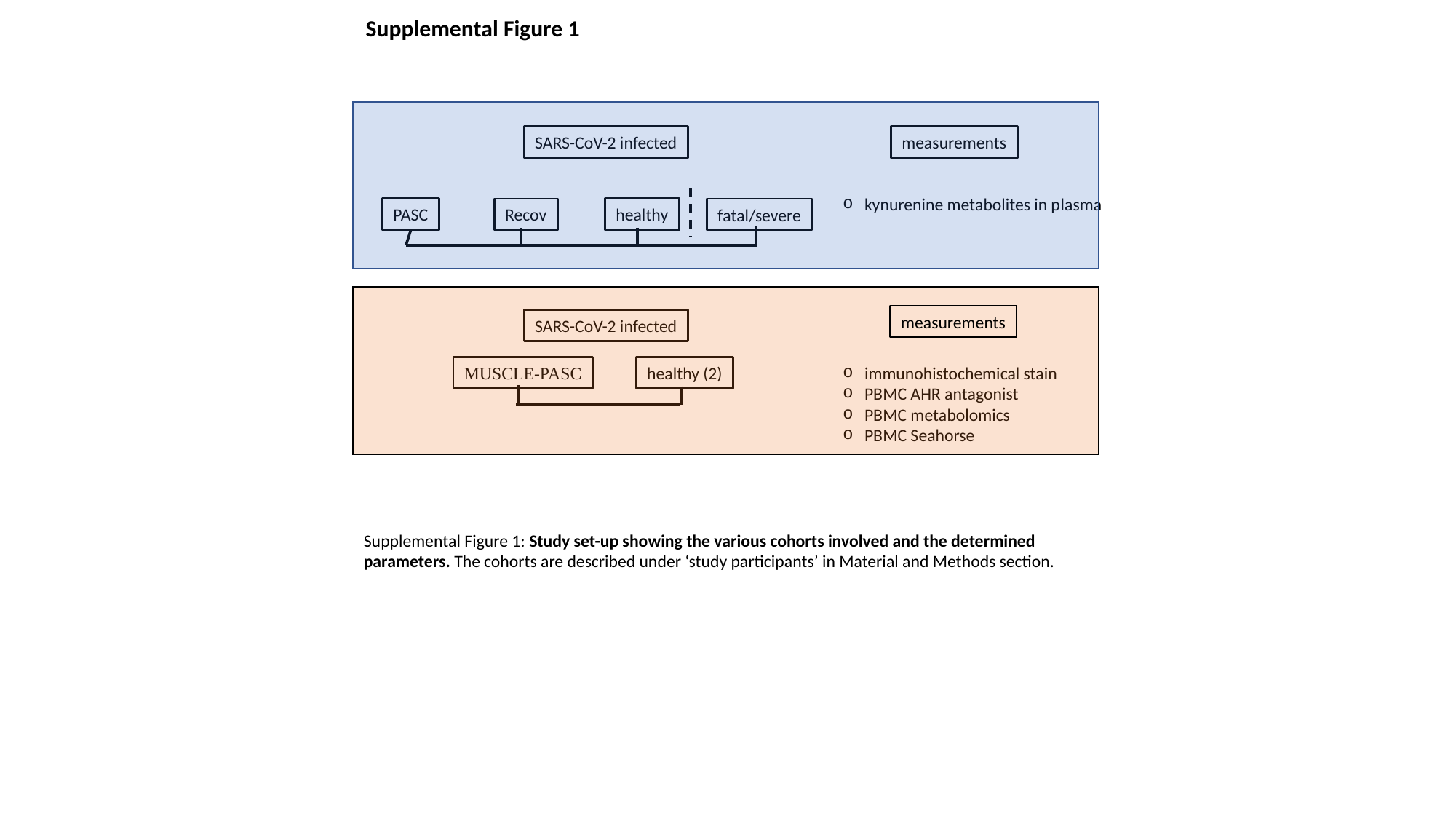

Supplemental Figure 1
SARS-CoV-2 infected
measurements
kynurenine metabolites in plasma
PASC
healthy
Recov
fatal/severe
measurements
SARS-CoV-2 infected
MUSCLE-PASC
healthy (2)
immunohistochemical stain
PBMC AHR antagonist
PBMC metabolomics
PBMC Seahorse
Supplemental Figure 1: Study set-up showing the various cohorts involved and the determined parameters. The cohorts are described under ‘study participants’ in Material and Methods section.

## Slide 2
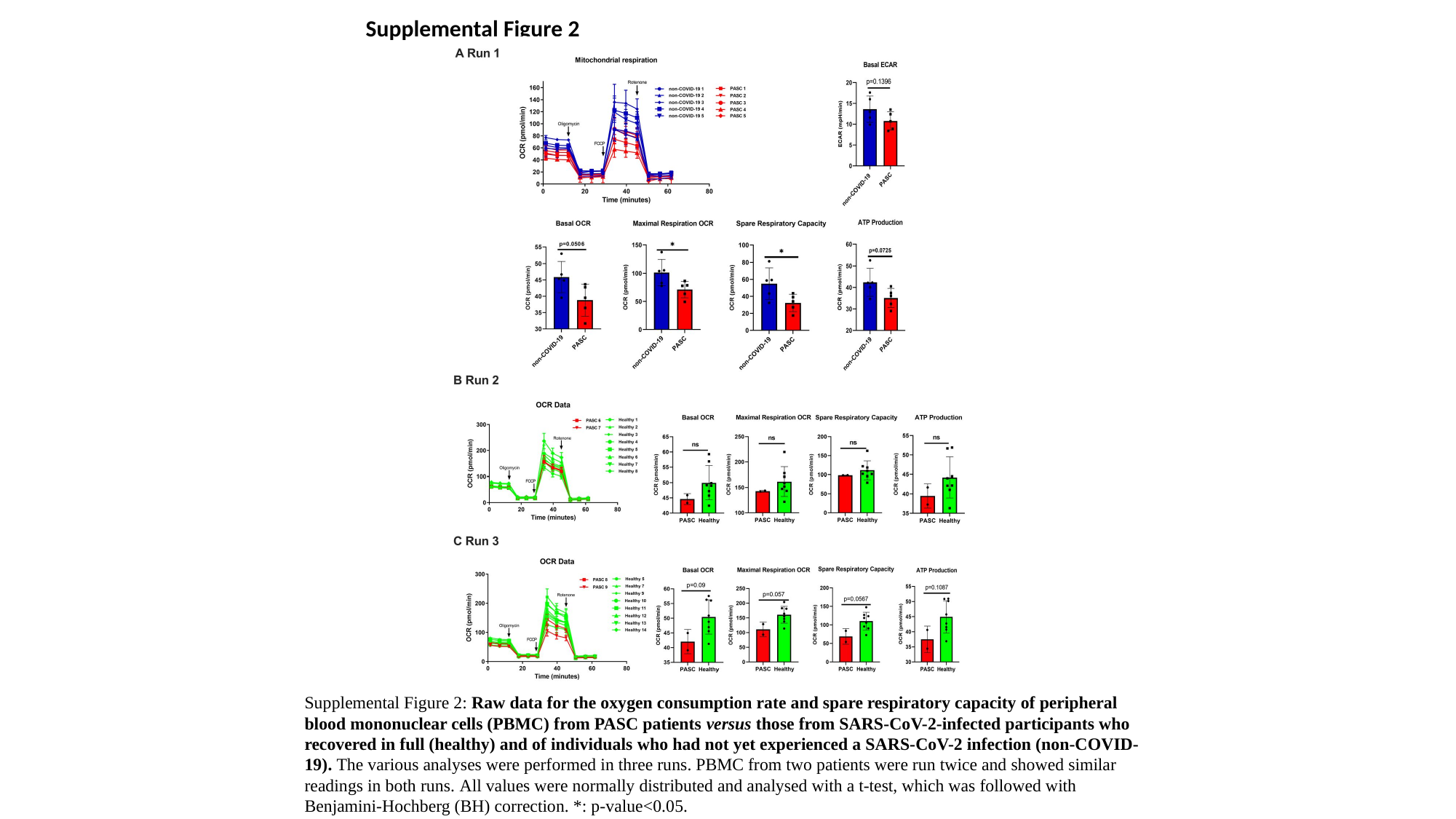

Supplemental Figure 2
Supplemental Figure 2: Raw data for the oxygen consumption rate and spare respiratory capacity of peripheral blood mononuclear cells (PBMC) from PASC patients versus those from SARS-CoV-2-infected participants who recovered in full (healthy) and of individuals who had not yet experienced a SARS-CoV-2 infection (non-COVID-19). The various analyses were performed in three runs. PBMC from two patients were run twice and showed similar readings in both runs. All values were normally distributed and analysed with a t-test, which was followed with Benjamini-Hochberg (BH) correction. *: p-value<0.05.

## Slide 3
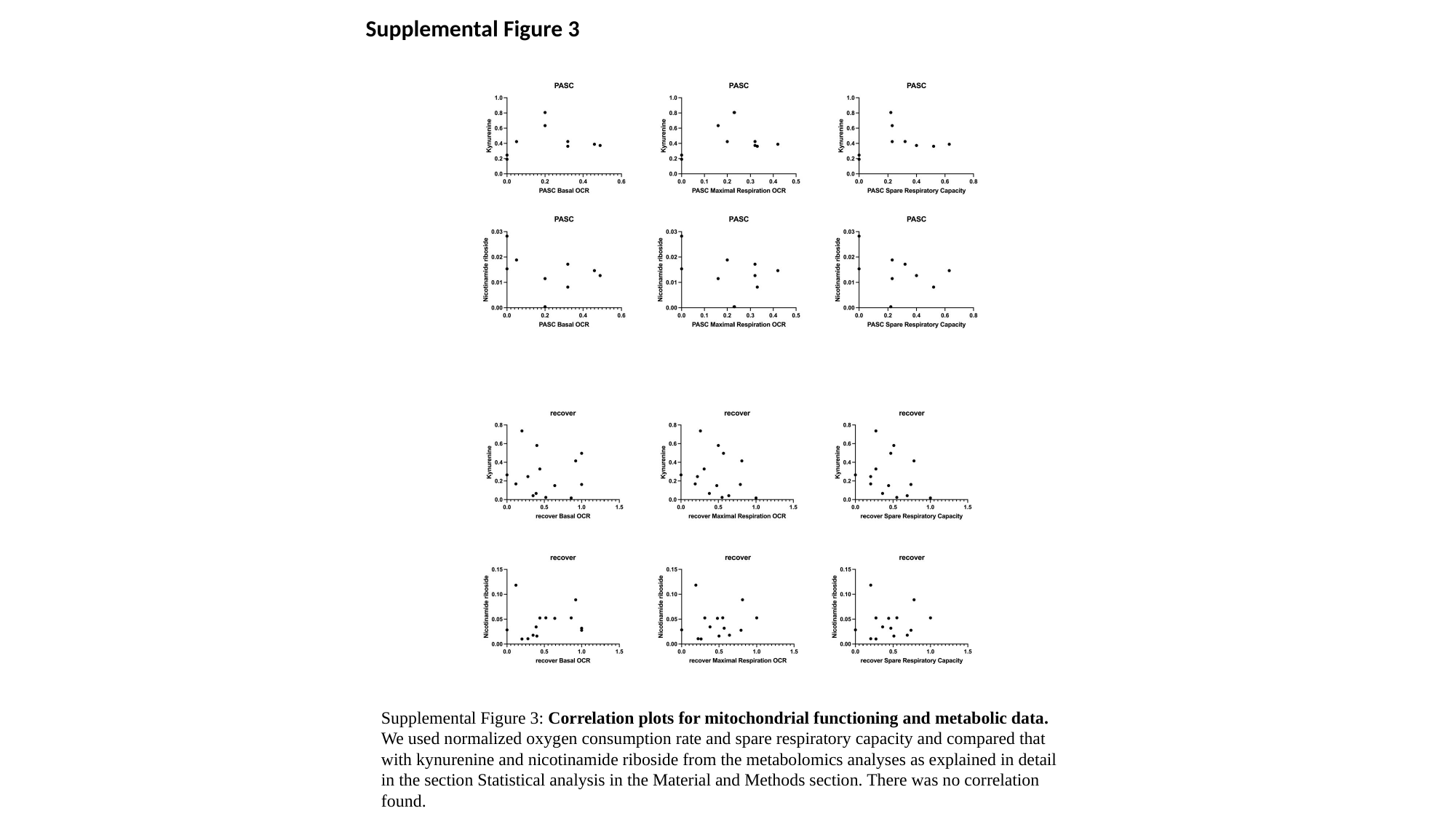

Supplemental Figure 3
Supplemental Figure 3: Correlation plots for mitochondrial functioning and metabolic data. We used normalized oxygen consumption rate and spare respiratory capacity and compared that with kynurenine and nicotinamide riboside from the metabolomics analyses as explained in detail in the section Statistical analysis in the Material and Methods section. There was no correlation found.

## Slide 4
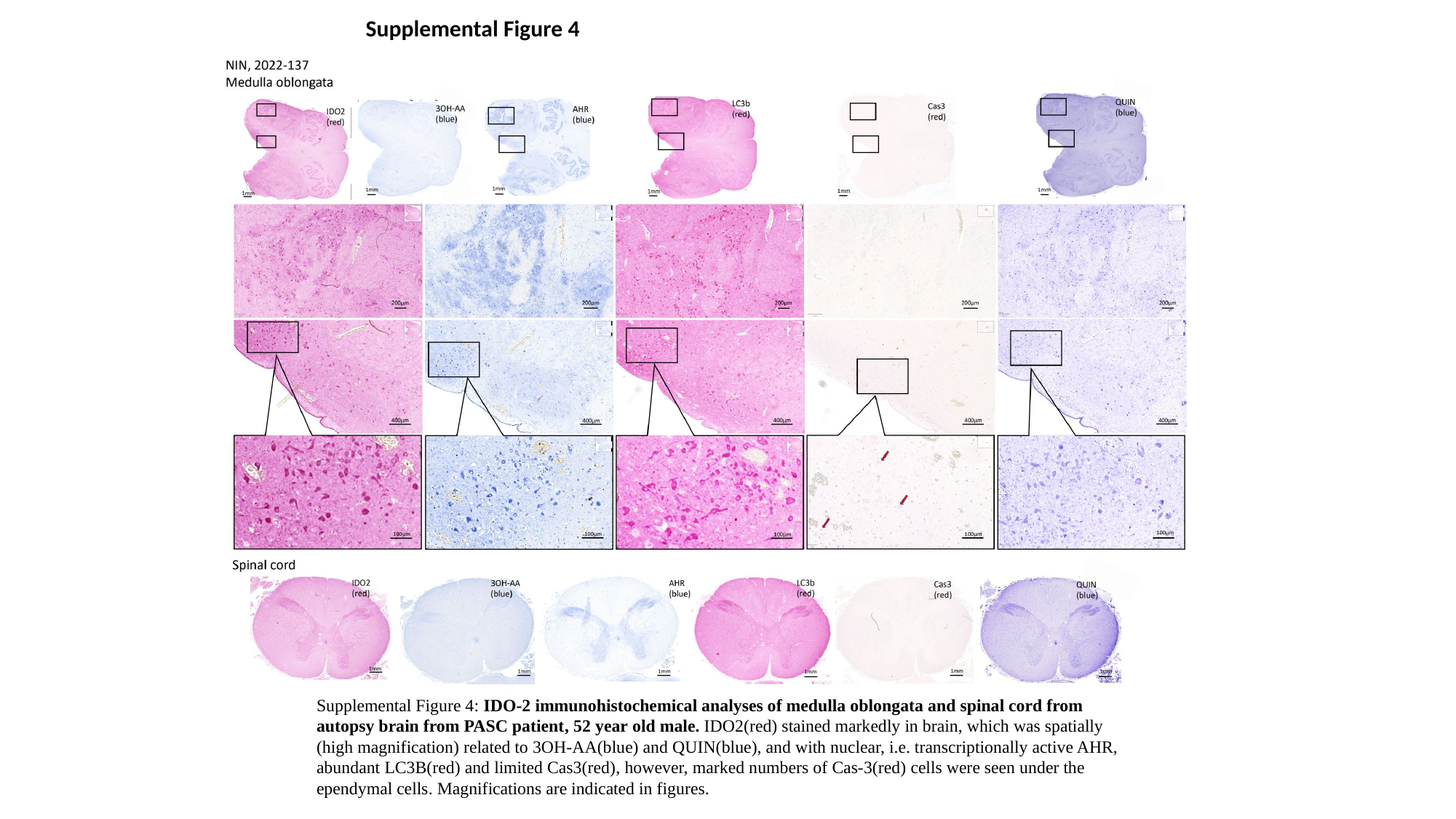

Supplemental Figure 4
Supplemental Figure 4: IDO-2 immunohistochemical analyses of medulla oblongata and spinal cord from autopsy brain from PASC patient, 52 year old male. IDO2(red) stained markedly in brain, which was spatially (high magnification) related to 3OH-AA(blue) and QUIN(blue), and with nuclear, i.e. transcriptionally active AHR, abundant LC3B(red) and limited Cas3(red), however, marked numbers of Cas-3(red) cells were seen under the ependymal cells. Magnifications are indicated in figures.

## Slide 5
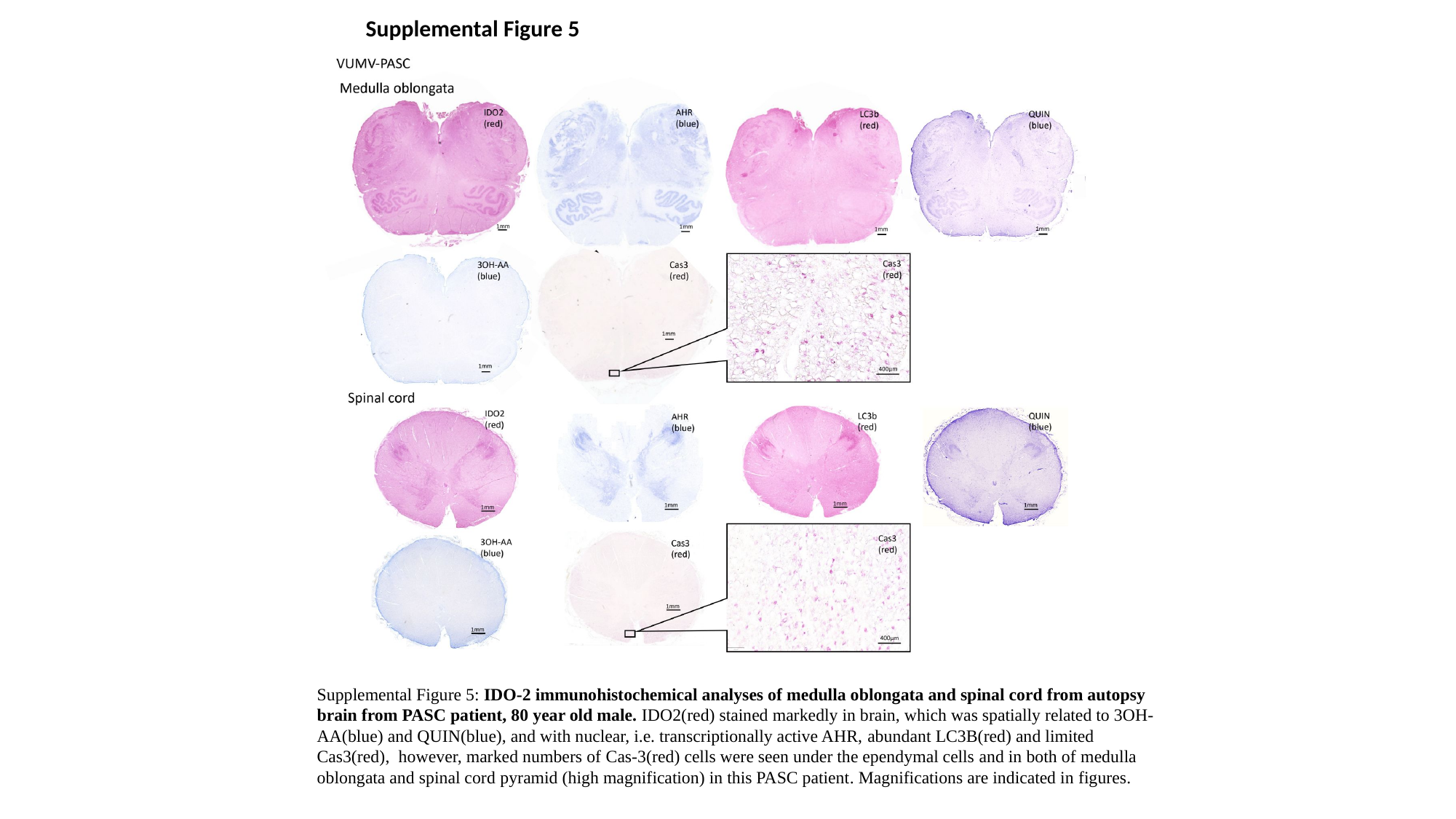

Supplemental Figure 5
Supplemental Figure 5: IDO-2 immunohistochemical analyses of medulla oblongata and spinal cord from autopsy brain from PASC patient, 80 year old male. IDO2(red) stained markedly in brain, which was spatially related to 3OH-AA(blue) and QUIN(blue), and with nuclear, i.e. transcriptionally active AHR, abundant LC3B(red) and limited Cas3(red), however, marked numbers of Cas-3(red) cells were seen under the ependymal cells and in both of medulla oblongata and spinal cord pyramid (high magnification) in this PASC patient. Magnifications are indicated in figures.

## Slide 6
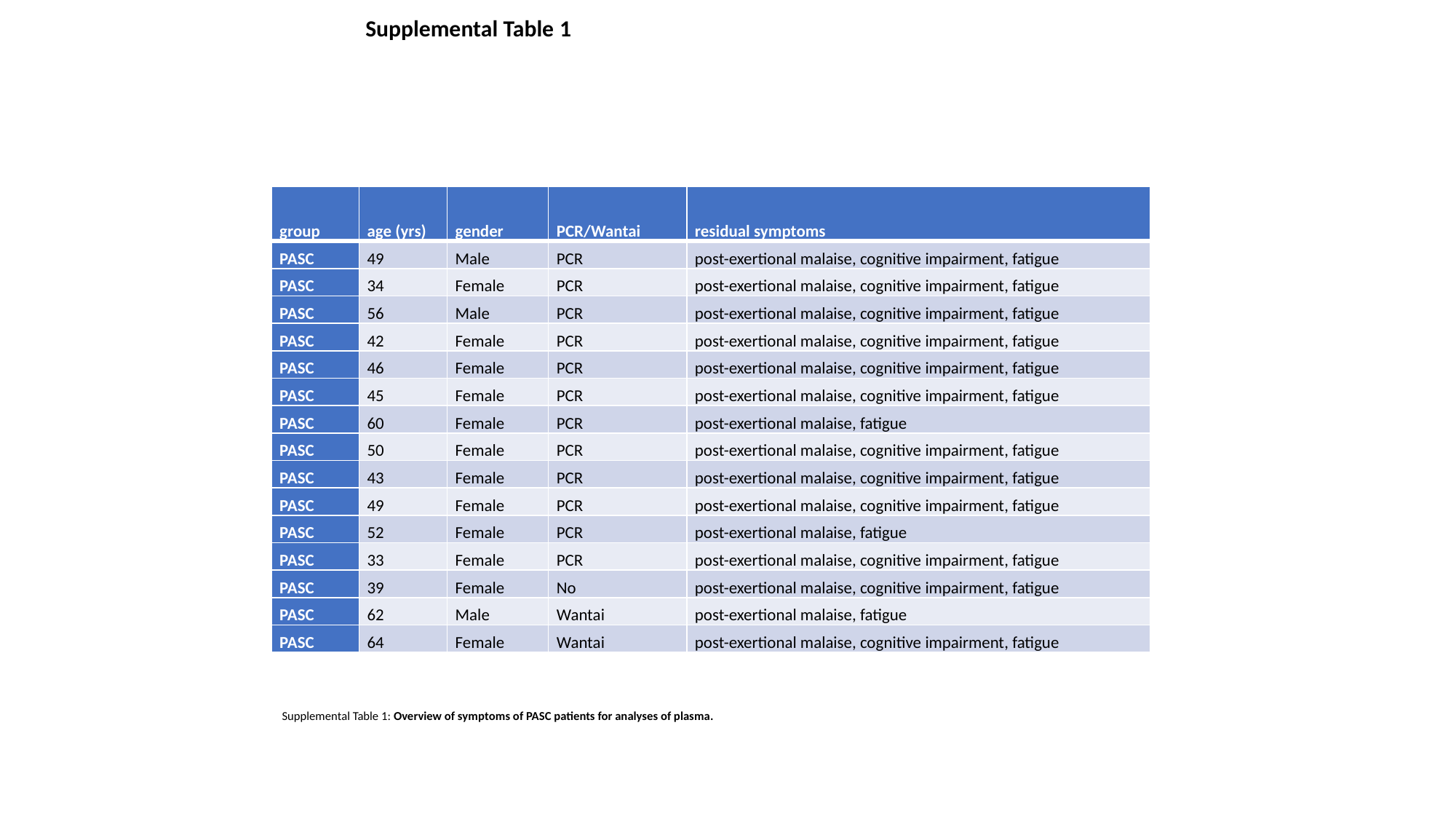

Supplemental Table 1
| group | age (yrs) | gender | PCR/Wantai | residual symptoms |
| --- | --- | --- | --- | --- |
| PASC | 49 | Male | PCR | post-exertional malaise, cognitive impairment, fatigue |
| PASC | 34 | Female | PCR | post-exertional malaise, cognitive impairment, fatigue |
| PASC | 56 | Male | PCR | post-exertional malaise, cognitive impairment, fatigue |
| PASC | 42 | Female | PCR | post-exertional malaise, cognitive impairment, fatigue |
| PASC | 46 | Female | PCR | post-exertional malaise, cognitive impairment, fatigue |
| PASC | 45 | Female | PCR | post-exertional malaise, cognitive impairment, fatigue |
| PASC | 60 | Female | PCR | post-exertional malaise, fatigue |
| PASC | 50 | Female | PCR | post-exertional malaise, cognitive impairment, fatigue |
| PASC | 43 | Female | PCR | post-exertional malaise, cognitive impairment, fatigue |
| PASC | 49 | Female | PCR | post-exertional malaise, cognitive impairment, fatigue |
| PASC | 52 | Female | PCR | post-exertional malaise, fatigue |
| PASC | 33 | Female | PCR | post-exertional malaise, cognitive impairment, fatigue |
| PASC | 39 | Female | No | post-exertional malaise, cognitive impairment, fatigue |
| PASC | 62 | Male | Wantai | post-exertional malaise, fatigue |
| PASC | 64 | Female | Wantai | post-exertional malaise, cognitive impairment, fatigue |
Supplemental Table 1: Overview of symptoms of PASC patients for analyses of plasma.

## Slide 7
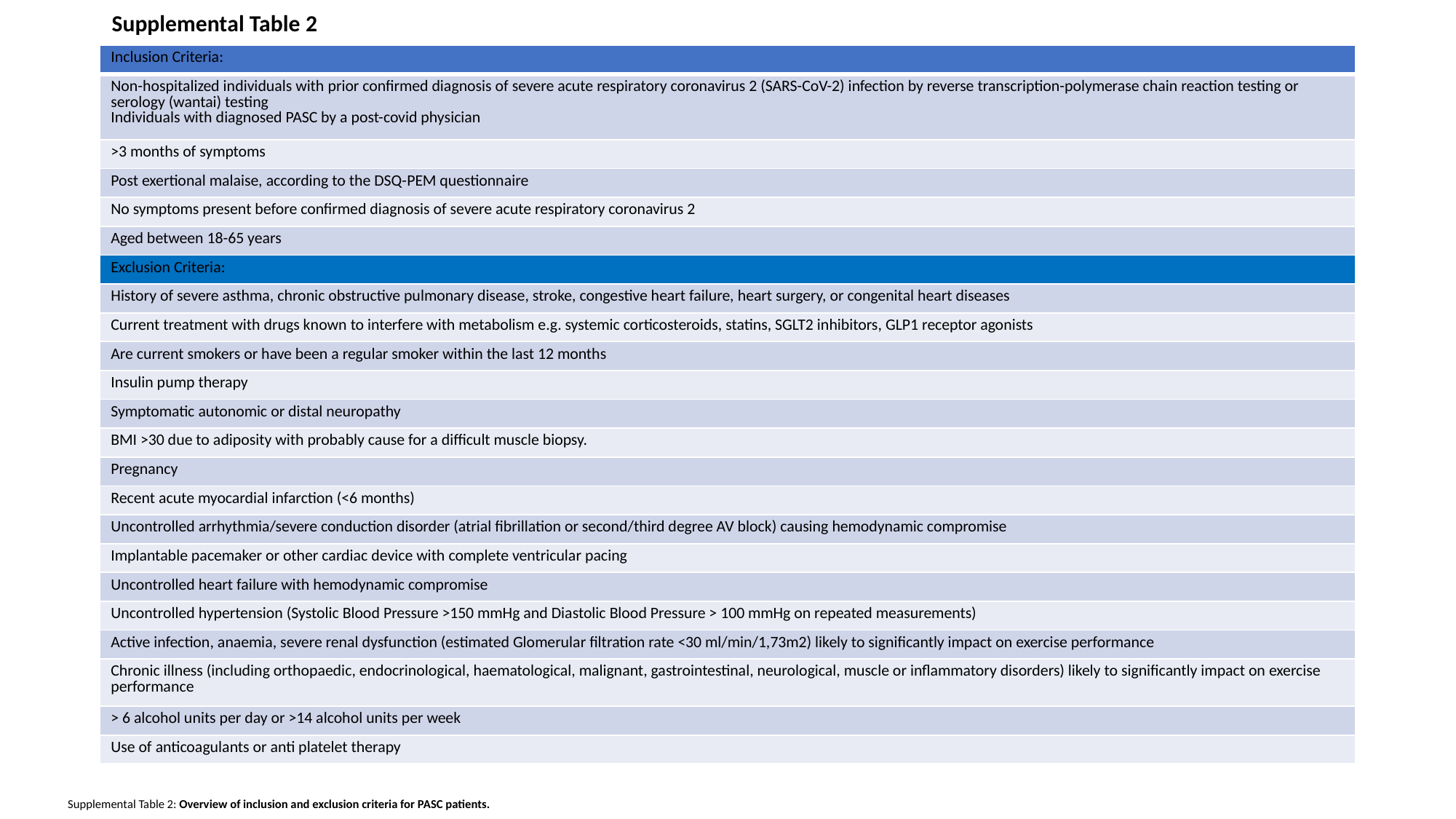

Supplemental Table 2
| Inclusion Criteria: |
| --- |
| Non-hospitalized individuals with prior confirmed diagnosis of severe acute respiratory coronavirus 2 (SARS-CoV-2) infection by reverse transcription-polymerase chain reaction testing or serology (wantai) testing Individuals with diagnosed PASC by a post-covid physician |
| >3 months of symptoms |
| Post exertional malaise, according to the DSQ-PEM questionnaire |
| No symptoms present before confirmed diagnosis of severe acute respiratory coronavirus 2 |
| Aged between 18-65 years |
| Exclusion Criteria: |
| History of severe asthma, chronic obstructive pulmonary disease, stroke, congestive heart failure, heart surgery, or congenital heart diseases |
| Current treatment with drugs known to interfere with metabolism e.g. systemic corticosteroids, statins, SGLT2 inhibitors, GLP1 receptor agonists |
| Are current smokers or have been a regular smoker within the last 12 months |
| Insulin pump therapy |
| Symptomatic autonomic or distal neuropathy |
| BMI >30 due to adiposity with probably cause for a difficult muscle biopsy. |
| Pregnancy |
| Recent acute myocardial infarction (<6 months) |
| Uncontrolled arrhythmia/severe conduction disorder (atrial fibrillation or second/third degree AV block) causing hemodynamic compromise |
| Implantable pacemaker or other cardiac device with complete ventricular pacing |
| Uncontrolled heart failure with hemodynamic compromise |
| Uncontrolled hypertension (Systolic Blood Pressure >150 mmHg and Diastolic Blood Pressure > 100 mmHg on repeated measurements) |
| Active infection, anaemia, severe renal dysfunction (estimated Glomerular filtration rate <30 ml/min/1,73m2) likely to significantly impact on exercise performance |
| Chronic illness (including orthopaedic, endocrinological, haematological, malignant, gastrointestinal, neurological, muscle or inflammatory disorders) likely to significantly impact on exercise performance |
| > 6 alcohol units per day or >14 alcohol units per week |
| Use of anticoagulants or anti platelet therapy |
Supplemental Table 2: Overview of inclusion and exclusion criteria for PASC patients.

## Slide 8
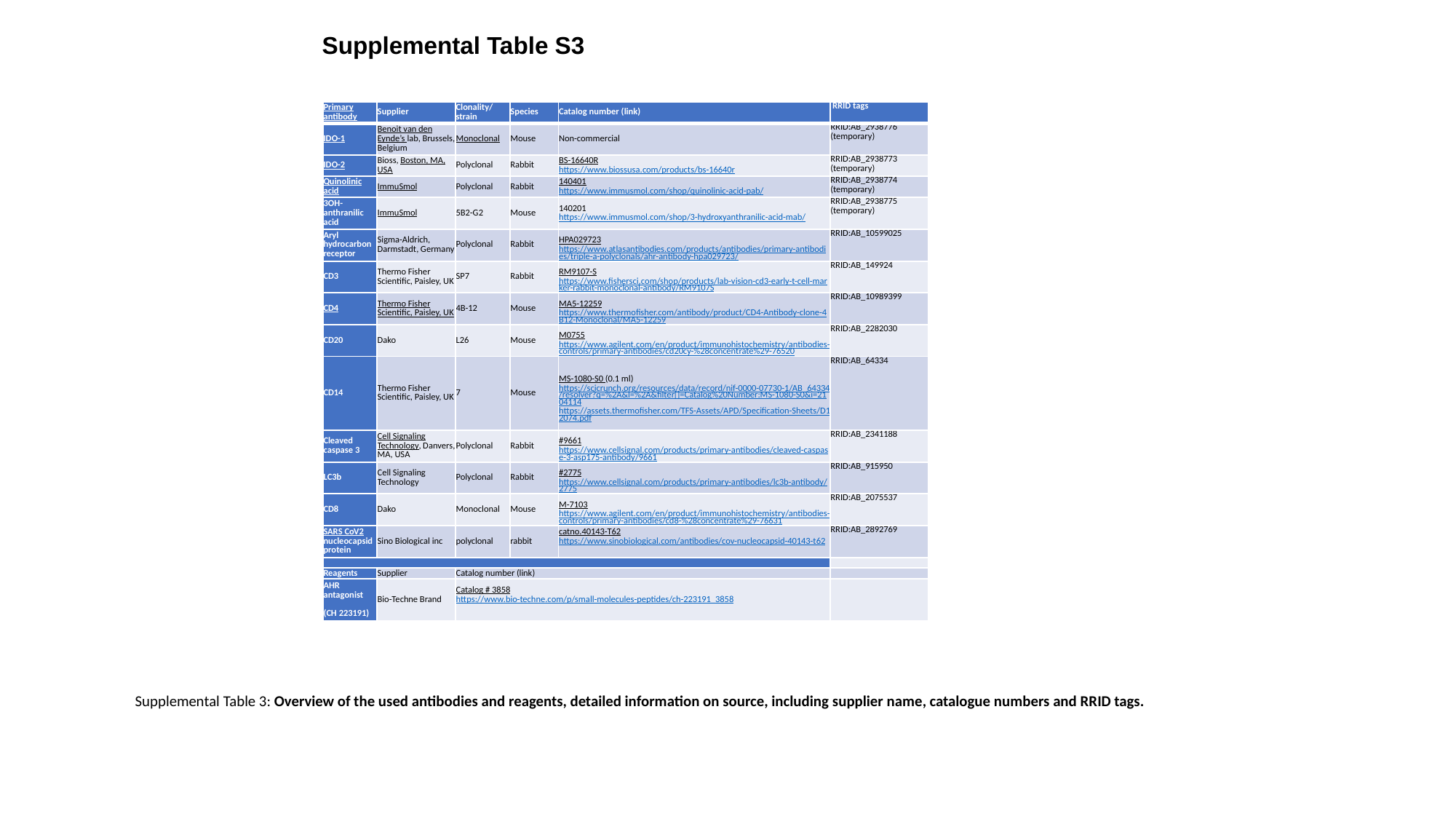

Supplemental Table S3
| Primary antibody | Supplier | Clonality/strain | Species | Catalog number (link) | RRID tags |
| --- | --- | --- | --- | --- | --- |
| IDO-1 | Benoit van den Eynde’s lab, Brussels, Belgium | Monoclonal | Mouse | Non-commercial | RRID:AB\_2938776 (temporary) |
| IDO-2 | Bioss, Boston, MA, USA | Polyclonal | Rabbit | BS-16640R https://www.biossusa.com/products/bs-16640r | RRID:AB\_2938773 (temporary) |
| Quinolinic acid | ImmuSmol | Polyclonal | Rabbit | 140401 https://www.immusmol.com/shop/quinolinic-acid-pab/ | RRID:AB\_2938774 (temporary) |
| 3OH-anthranilic acid | ImmuSmol | 5B2-G2 | Mouse | 140201 https://www.immusmol.com/shop/3-hydroxyanthranilic-acid-mab/ | RRID:AB\_2938775 (temporary) |
| Aryl hydrocarbon receptor | Sigma-Aldrich, Darmstadt, Germany | Polyclonal | Rabbit | HPA029723 https://www.atlasantibodies.com/products/antibodies/primary-antibodies/triple-a-polyclonals/ahr-antibody-hpa029723/ | RRID:AB\_10599025 |
| CD3 | Thermo Fisher Scientific, Paisley, UK | SP7 | Rabbit | RM9107-S https://www.fishersci.com/shop/products/lab-vision-cd3-early-t-cell-marker-rabbit-monoclonal-antibody/RM9107S | RRID:AB\_149924 |
| CD4 | Thermo Fisher Scientific, Paisley, UK | 4B-12 | Mouse | MA5-12259 https://www.thermofisher.com/antibody/product/CD4-Antibody-clone-4B12-Monoclonal/MA5-12259 | RRID:AB\_10989399 |
| CD20 | Dako | L26 | Mouse | M0755 https://www.agilent.com/en/product/immunohistochemistry/antibodies-controls/primary-antibodies/cd20cy-%28concentrate%29-76520 | RRID:AB\_2282030 |
| CD14 | Thermo Fisher Scientific, Paisley, UK | 7 | Mouse | MS-1080-S0 (0.1 ml) https://scicrunch.org/resources/data/record/nif-0000-07730-1/AB\_64334/resolver?q=%2A&l=%2A&filter[]=Catalog%20Number:MS-1080-S0&i=2104114 https://assets.thermofisher.com/TFS-Assets/APD/Specification-Sheets/D12074.pdf | RRID:AB\_64334 |
| Cleaved caspase 3 | Cell Signaling Technology, Danvers, MA, USA | Polyclonal | Rabbit | #9661 https://www.cellsignal.com/products/primary-antibodies/cleaved-caspase-3-asp175-antibody/9661 | RRID:AB\_2341188 |
| LC3b | Cell Signaling Technology | Polyclonal | Rabbit | #2775 https://www.cellsignal.com/products/primary-antibodies/lc3b-antibody/2775 | RRID:AB\_915950 |
| CD8 | Dako | Monoclonal | Mouse | M-7103 https://www.agilent.com/en/product/immunohistochemistry/antibodies-controls/primary-antibodies/cd8-%28concentrate%29-76631 | RRID:AB\_2075537 |
| SARS CoV2 nucleocapsid protein | Sino Biological inc | polyclonal | rabbit | catno.40143-T62 https://www.sinobiological.com/antibodies/cov-nucleocapsid-40143-t62 | RRID:AB\_2892769 |
| | | | | | |
| Reagents | Supplier | Catalog number (link) | | | |
| AHR antagonist   (CH 223191) | Bio-Techne Brand | Catalog # 3858 https://www.bio-techne.com/p/small-molecules-peptides/ch-223191\_3858 | | | |
Supplemental Table 3: Overview of the used antibodies and reagents, detailed information on source, including supplier name, catalogue numbers and RRID tags.

## Slide 9
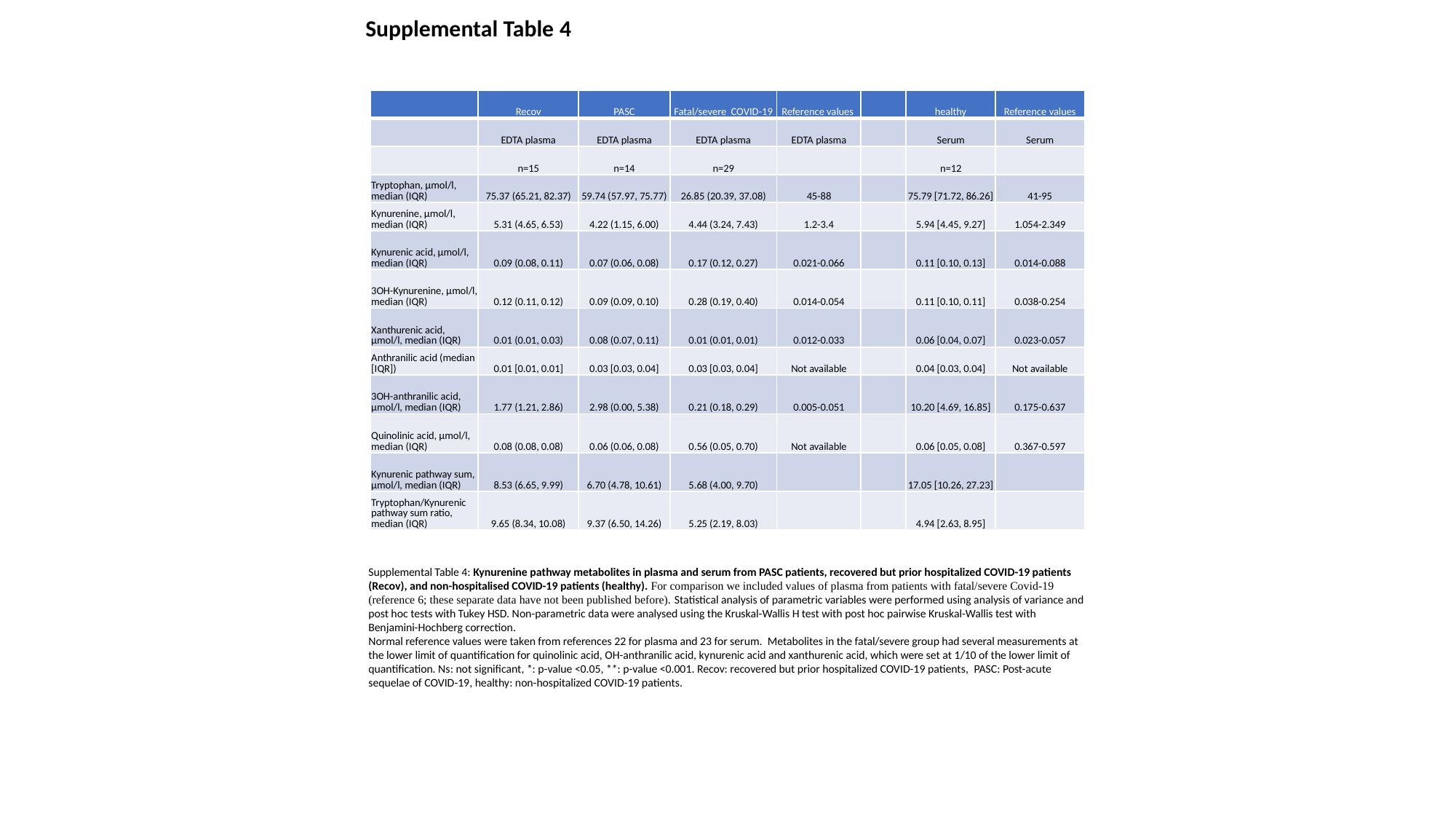

Supplemental Table 4
| | Recov | PASC | Fatal/severe COVID-19 | Reference values | | healthy | Reference values |
| --- | --- | --- | --- | --- | --- | --- | --- |
| | EDTA plasma | EDTA plasma | EDTA plasma | EDTA plasma | | Serum | Serum |
| | n=15 | n=14 | n=29 | | | n=12 | |
| Tryptophan, µmol/l, median (IQR) | 75.37 (65.21, 82.37) | 59.74 (57.97, 75.77) | 26.85 (20.39, 37.08) | 45-88 | | 75.79 [71.72, 86.26] | 41-95 |
| Kynurenine, µmol/l, median (IQR) | 5.31 (4.65, 6.53) | 4.22 (1.15, 6.00) | 4.44 (3.24, 7.43) | 1.2-3.4 | | 5.94 [4.45, 9.27] | 1.054-2.349 |
| Kynurenic acid, µmol/l, median (IQR) | 0.09 (0.08, 0.11) | 0.07 (0.06, 0.08) | 0.17 (0.12, 0.27) | 0.021-0.066 | | 0.11 [0.10, 0.13] | 0.014-0.088 |
| 3OH-Kynurenine, µmol/l, median (IQR) | 0.12 (0.11, 0.12) | 0.09 (0.09, 0.10) | 0.28 (0.19, 0.40) | 0.014-0.054 | | 0.11 [0.10, 0.11] | 0.038-0.254 |
| Xanthurenic acid, µmol/l, median (IQR) | 0.01 (0.01, 0.03) | 0.08 (0.07, 0.11) | 0.01 (0.01, 0.01) | 0.012-0.033 | | 0.06 [0.04, 0.07] | 0.023-0.057 |
| Anthranilic acid (median [IQR]) | 0.01 [0.01, 0.01] | 0.03 [0.03, 0.04] | 0.03 [0.03, 0.04] | Not available | | 0.04 [0.03, 0.04] | Not available |
| 3OH-anthranilic acid, µmol/l, median (IQR) | 1.77 (1.21, 2.86) | 2.98 (0.00, 5.38) | 0.21 (0.18, 0.29) | 0.005-0.051 | | 10.20 [4.69, 16.85] | 0.175-0.637 |
| Quinolinic acid, µmol/l, median (IQR) | 0.08 (0.08, 0.08) | 0.06 (0.06, 0.08) | 0.56 (0.05, 0.70) | Not available | | 0.06 [0.05, 0.08] | 0.367-0.597 |
| Kynurenic pathway sum, µmol/l, median (IQR) | 8.53 (6.65, 9.99) | 6.70 (4.78, 10.61) | 5.68 (4.00, 9.70) | | | 17.05 [10.26, 27.23] | |
| Tryptophan/Kynurenic pathway sum ratio, median (IQR) | 9.65 (8.34, 10.08) | 9.37 (6.50, 14.26) | 5.25 (2.19, 8.03) | | | 4.94 [2.63, 8.95] | |
Supplemental Table 4: Kynurenine pathway metabolites in plasma and serum from PASC patients, recovered but prior hospitalized COVID-19 patients (Recov), and non-hospitalised COVID-19 patients (healthy). For comparison we included values of plasma from patients with fatal/severe Covid-19 (reference 6; these separate data have not been published before). Statistical analysis of parametric variables were performed using analysis of variance and post hoc tests with Tukey HSD. Non-parametric data were analysed using the Kruskal-Wallis H test with post hoc pairwise Kruskal-Wallis test with Benjamini-Hochberg correction.
Normal reference values were taken from references 22 for plasma and 23 for serum.  Metabolites in the fatal/severe group had several measurements at the lower limit of quantification for quinolinic acid, OH-anthranilic acid, kynurenic acid and xanthurenic acid, which were set at 1/10 of the lower limit of quantification. Ns: not significant, *: p-value <0.05, **: p-value <0.001. Recov: recovered but prior hospitalized COVID-19 patients,  PASC: Post-acute sequelae of COVID-19, healthy: non-hospitalized COVID-19 patients.
